# Mucinous cystic neoplasms of the pancreas and liver share a similar DNA methylation profile with mucinous ovarian tumors

**DOI:** 10.1002/path.6439

**Published:** 2025-06-25

**Authors:** Zoe Leoni, Teodor G Calina, Tobias Janik, Elena Grafenhorst, Eliane T Taube, Christopher CM Neumann, BaoQing Chen, Elena I Braicu, Jalid Sehouli, Thomas Malinka, Wenzel Schöning, Johann Pratschke, George A Calin, David S Klimstra, Jamal K Benhamida, Irene Esposito, Markus Möbs, David Horst, Simon Schallenberg, David Capper, Mihnea P Dragomir

**Affiliations:** ^1^ Institute of Pathology, Charité – Universitätsmedizin Berlin, Corporate Member of Freie Universität Berlin Humboldt‐Universität zu Berlin Berlin Germany; ^2^ Faculty of Physics Babeș‐Bolyai University Cluj‐Napoca Romania; ^3^ TGC Ventures UG Berlin Germany; ^4^ German Cancer Consortium (DKTK) Partner Site Berlin, and German Cancer Research Center (DKFZ) Heidelberg Germany; ^5^ Department of Hematology, Oncology and Tumor Immunology, Charité – Universitätsmedizin Berlin, Corporate Member of Freie Universität Berlin Humboldt‐Universität zu Berlin Berlin Germany; ^6^ Department of Radiation Oncology, State Key Laboratory of Oncology in South China, Guangdong Provincial Clinical Research Center for Cancer Sun Yat‐sen University Cancer Center Guangzhou PR China; ^7^ Guangdong Esophageal Cancer Research Institute Guangzhou PR China; ^8^ Department of Gynaecology, European Competence Center for Ovarian Cancer Charité – Universitätsmedizin Berlin, Corporate Member of Freie Universität Berlin Humboldt‐Universität zu Berlin Berlin Germany; ^9^ North Eastern German Society for Gynecological Cancer Tumor Bank Ovarian Cancer Network Berlin Germany; ^10^ Department of Surgery, Experimental Surgery, CCM, CVK, Charité – Universitätsmedizin Berlin, Corporate Member of Freie Universität Berlin Humboldt‐Universität zu Berlin Berlin Germany; ^11^ Department of Translational Molecular Pathology The University of Texas MD Anderson Cancer Center Houston TX USA; ^12^ The Non‐coding RNA Center The University of Texas MD Anderson Cancer Center Houston TX USA; ^13^ Department of Pathology Yale School of Medicine New Haven CT USA; ^14^ Department of Pathology and Laboratory Medicine Memorial Sloan Kettering Cancer Center New York NY USA; ^15^ Institute of Pathology Heinrich‐Heine‐University and University Hospital of Düsseldorf Düsseldorf Germany; ^16^ Department of Neuropathology, Charité – Universitätsmedizin Berlin, Corporate Member of Freie Universität Berlin Humboldt‐Universität zu Berlin Berlin Germany; ^17^ Berlin Institute of Health at Charité – Universitätsmedizin Berlin Berlin Germany

**Keywords:** mucinous cystic neoplasms, DNA methylation, mucinous ovarian carcinoma, mucinous borderline ovarian tumor

## Abstract

The origin of mucinous cystic neoplasms (MCNs) remains a major challenge in hepato‐pancreato‐biliary pathology. These cystic tumors are defined by their mucinous epithelium and ovarian‐like stroma, with an estimated 10% risk of progression to invasive carcinoma. The origin of the ovarian‐like stroma remains a subject of debate. In this study, we conducted immunohistochemical profiling, targeted DNA sequencing, and genome‐wide DNA methylation analysis on a cohort of 15 pancreatic MCNs (MCN‐P) and six hepatic MCNs (MCN‐L). Using immunohistochemistry and targeted DNA sequencing, we unequivocally established the diagnosis of MCN. Unsupervised DNA methylation profile analysis of reference classes of pancreatic neoplasms (11 entities and normal pancreatic tissue from 224 unique samples) revealed that MCN‐P predominantly forms a distinct group. In the DNA methylation landscape of liver tumors, encompassing five tumor types and normal bile duct tissue from 136 unique samples, MCN‐L demonstrated a specific methylation profile when compared with all other entities. Furthermore, within the DNA methylation landscape of ovarian tumors – featuring five tumor types, normal Fallopian tube, and normal ovarian tissue from 90 unique samples – we found that both MCN‐P and MCN‐L grouped with mucinous ovarian carcinoma and mucinous borderline ovarian tumors (mBOTs). Notably, low‐grade MCNs exhibited greater DNA methylation similarities to mBOTs, while high‐grade or invasive MCNs were primarily associated with mucinous ovarian carcinomas. When analyzing all samples together (19 tumor types and four normal tissue types, *n* = 430), MCNs similarly grouped with mucinous ovarian tumors and normal ovarian tissue. Additionally, in a network analysis of differentially methylated probes, MCN‐P and MCN‐L share significant methylation traits, closely resembling mucinous ovarian tumors. In conclusion, our findings highlight that MCN‐P and MCN‐L are distinct entities in the landscape of pancreatic and hepatic tumors and show DNA methylation profile similarities with mucinous ovarian tumors, suggesting a potential common origin. © 2025 The Author(s). *The Journal of Pathology* published by John Wiley & Sons Ltd on behalf of The Pathological Society of Great Britain and Ireland.

## Introduction

Mucinous cystic neoplasms (MCNs) of the pancreas (MCN‐P) and liver (MCN‐L) are rare cystic lesions characterized by a mucinous epithelium and an ovarian‐like stroma. These cysts do not directly communicate with the ductal system. MCNs are generally low‐grade lesions that can progress to high‐grade lesions and, in approximately 10% of cases, to invasive pancreatic ductal adenocarcinoma (PDAC) and intrahepatic cholangiocarcinoma (iCCA) [1].

The origin of these neoplasms remains unclear. Epidemiologically, these entities occur almost exclusively in women (mean age at diagnosis is 40–50 years), and MCN‐P are specifically localized in the body and tail of the pancreas. These observations and the specific ovarian‐like stroma suggest possible similarities with mucinous ovarian tumors. In addition, MCNs and mucinous ovarian tumors have been shown to be driven by *KRAS* and *RNF43* mutations in similar proportions [2, 3]. Recent data have confirmed that mucinous ovarian neoplasms originate from mucinous cystadenomas, which may progress to mucinous borderline ovarian tumors (mBOTs) and in very few cases further evolve to primary mucinous ovarian carcinomas (PMOCs) [4], but the cell of origin of these neoplasms remains unknown. Furthermore, in a review paper, the same research group proposes conducting epigenetic analyses of MCN and mBOT/PMOC to better understand the origin of the latter [5].

Current theories suggest that MCN‐P and MCN‐L may originate from remnants of developing gonads during embryogenesis. Indeed, gene expression analysis data suggest that both MCNs and mucinous ovarian tumors originate from primordial germ cells and should be classified as germ cell tumor variants [6].

Genome‐wide DNA methylation patterns typically reveal the tissue of origin of various tumors and have recently been suggested as a potential method for characterizing poorly defined entities. This has been particularly interesting for metastatic tumors with an unknown primary [7, 8] since DNA methylation analysis, which can identify the primary tumor, could lead to a targeted therapeutic approach and potentially increase patients’ overall survival [9]. Therefore, we hypothesized that genome‐wide DNA methylation analysis of MCN‐P and MCN‐L and their comparison with mBOT and PMOC would provide additional insights into the origin of these entities. To test this hypothesis, we analyzed and compared the DNA methylation profile of MCN‐P with most types of pancreatic tumors; of MCN‐L with diverse liver tumors; and both MCN‐P and MCN‐L together with ovarian tumors.

## Materials and methods

### Ethics approval and consent to participate

The study was approved by the ethics commissions of Charité – Universitätsmedizin Berlin (EA1/079/22 and EA1/110/22). Formalin‐fixed paraffin‐embedded (FFPE) tissue from patients was acquired with informed consent following the local institutional review and the Declaration of Helsinki.

### Patient study cohort

We retrospectively analyzed the archive of the Institute of Pathology, Charité – Universitätsmedizin Berlin and retrieved all samples from 2006 to 2023 that included the term mucinous cystic neoplasm. We obtained FFPE blocks and corresponding slides for all specimens. All samples were reviewed by three pathologists (MPD, ZL, and SS), resulting in 15 MCN‐P and six MCN‐L. All samples were collected from female patients. DNA methylation analysis of these samples was performed using the Illumina Infinium™ MethylationEPIC v1.0 BeadChip array (Illumina, San Diego, CA, USA). Detailed methods for immunohistochemistry, DNA extraction, DNA methylation analysis and array processing, targeted next‐generation sequencing, unsupervised clustering (hierarchical and agglomerative consensus), pathway and cell type analysis, and decomposition of DNA methylated data using single‐cell RNA sequencing are provided in Supplementary materials and methods as well as in supplementary material, Tables S1 and S2.

To characterize the spectrum of pancreatic neoplasms, we included the following samples: 10 PDAC, 13 acinar cell carcinoma (ACC), 13 pancreatoblastoma (PB), 38 pancreatic neuroendocrine tumor (NET), 10 neuroendocrine carcinoma (NEC), 12 solid pseudopapillary neoplasm (SPN), and 9 normal pancreatic tissue samples from Benhamida *et al* [10]; 16 PDAC previously published in‐house samples from Dragomir *et al* [7]; one in‐house gastric‐type intraductal papillary mucinous neoplasm (gIPMN); and 32 gIPMN, 27 pancreatic intraepithelial neoplasia (PanIN), and 20 intestinal IPMN (iIPMN) samples from Liffers *et al* [11]. All pancreatic samples were derived from FFPE tissue.

To characterize the range of liver neoplasms, we included the following samples: 36 iCCA in‐house samples from Dragomir *et al* [7]; 19 hepatocellular carcinoma (HCC) samples from Cerapio *et al* [12]; and 50 normal bile duct, 9 iCCA, 8 intrahepatic intraductal papillary neoplasia of the bile duct (iIPNB), and 8 intrahepatic intraductal tubulopapillary neoplasia of the bile duct (iITPN) samples from Goeppert *et al* [13]. The samples from Dragomir *et al* [7], and Goeppert *et al* [13] were derived from FFPE tissue, while the samples from Cerapio *et al* [12] were fresh frozen.

To characterize the spectrum of ovarian neoplasms, we used the following samples: 16 high‐grade serous ovarian carcinoma (HGSOC) samples from Monjé *et al* [14]; 16 primary mucinous ovarian carcinoma (PMOC), 16 normal ovary, and 10 mBOT in‐house samples; and 12 normal Fallopian tube samples from Gonzalez Bosquet *et al* [15]. All samples were FFPE except for the 12 normal Fallopian tube samples, which were fresh frozen. For consistency, all the external samples analyzed were scanned using the Illumina Infinium™ MethylationEPIC v1.0 BeadChip array.

### Differentially methylated probes and construction of differentially methylated networks

The differentially methylated CpG probes (DMPs) between all 23 sample groups included in this study were determined using the limma R package as implemented in ChAMP [16]. CpGs with an adjusted *p* value less than 0.01 and an absolute log fold‐change (FC) value greater than 0.3 were considered differentially methylated between groups. This approach identified DMPs across 253 pairs of sample groups. Based on these data, relatedness networks were constructed using five different thresholds (0 CpGs, 1–999 CpGs, 1,000–4,999 CpGs, 5,000–9,999 CpGs, and 10,000–14,999 CpGs). A lower number of differentially methylated probes represents a stronger similarity between sample groups. In the network, nodes represent sample groups and edges correspond to the number of DMPs as defined by the thresholds. Additionally, for sample groups that remained disconnected from others, the closest possible connection was depicted, specifying the exact number of DMPs.

### Statistical analyses

Statistical analyses were performed using GraphPad Prism 9.0 (GraphPad Software, Boston, MA, USA). To compare H‐score levels between sample groups, data distribution was first evaluated using the Shapiro–Wilk normality test. For continuous variables, *p* values were calculated using an unpaired *t*‐test for normally distributed data, and the Mann–Whitney test for values with a non‐normal distribution. Comparisons of categorical variables (grade and mutational status) between groups were performed using the *χ* ^2^ test. Two‐sided *p* values less than 0.05 were considered statistically significant.

## Results

### Immunohistochemical and genetic profile of mucinous cystic neoplasm

**Figure 1:**
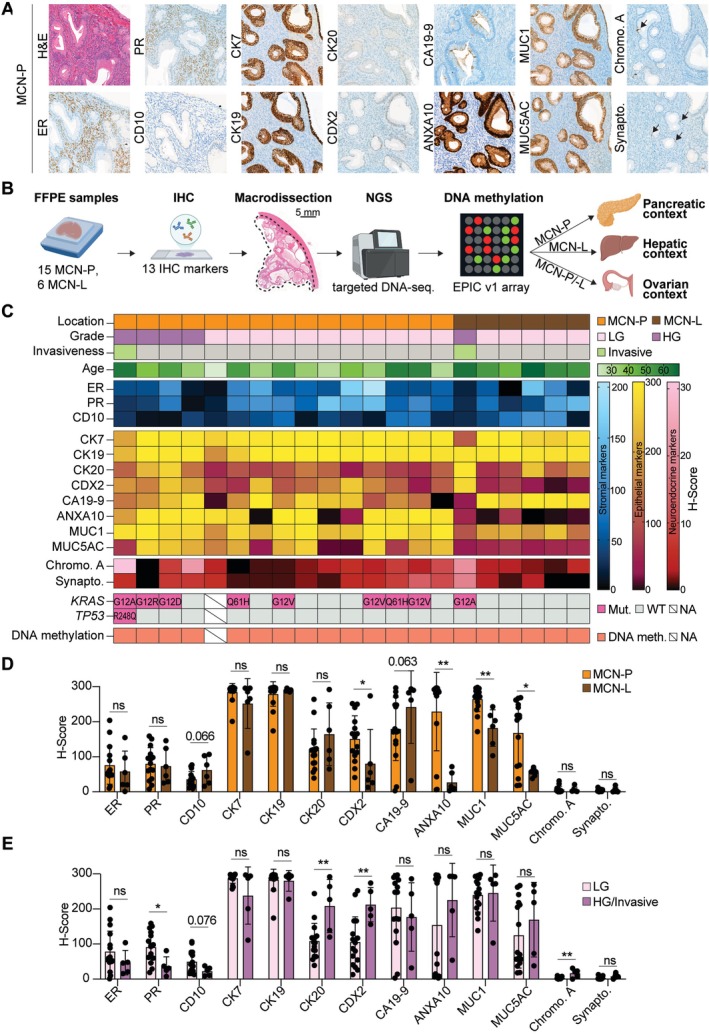
Immunohistochemical and genetic profile of mucinous cystic neoplasm of the pancreas and liver. (A) Representative H&E (20×) and IHC (20×) staining of a representative MCN‐P sample (case 12). Arrows indicate chromogranin A and synaptophysin positive cells. (B) Schematic overview of the study design. (C) H‐scores for stromal and epithelial markers of all samples. (D) Comparison of IHC expression profiles between MCN‐P and MCN‐L samples. (E) Comparison of IHC expression profiles between low‐grade (LG) and high‐grade (HG)/invasive samples. ER, estrogen receptor; PR, progesterone receptor; CD10, cluster of differentiation 10; CK7, cytokeratin 7; CK19, cytokeratin 19; CK20, cytokeratin 20; CDX2, caudal type homeobox 2; CA19‐9, carbohydrate antigen 19‐9; ANXA10, annexin A10; MUC1, mucin 1; MUC5AC, mucin 5AC; Chromo. A, chromogranin A; Synapto., synaptophysin; ns, not significant. *p < 0.05; **p < 0.01; ***p < 0.001; ****p < 0.0001. Panel B was created using BioRender.com.

To gain a deeper understanding of the origin of MCN‐P and MCN‐L, we performed a comprehensive IHC characterization of MCN‐P and MCN‐L (Figure 1A). All samples included in the study were evaluated by three pathologists independently, and the presence of ovarian‐like stroma was confirmed immunohistochemically. Representative images of progesterone receptor (PR) in stroma for all samples can be found in supplementary material, Figure S1.

To isolate the MCN epithelium and ovarian‐like stroma components only, while avoiding contamination from normal tissue, we carefully macrodissected sequential FFPE slides. Slides containing high‐grade and invasive components, if present, were included in the macrodissection. The macrodissected tissue was used for targeted DNA sequencing and genome‐wide DNA methylation analysis using the Illumina Infinium™ MethylationEPIC v1.0 BeadChip array. We compared the DNA methylation profile of MCN‐P with those of other pancreatic tumors, then that of MCN‐L with those of other hepatic tumors, and finally the profiles of both MCN‐P and MCN‐L with those of ovarian tumors (Figure 1B).

For the final analysis, we included 21 samples (supplementary material, Table S3): 15 MCN‐P and 6 MCN‐L. All MCN‐P samples were localized in the body and/or tail of the pancreas. Four MCN‐P samples showed high‐grade features, with one sample displaying invasive growth. All but one of the MCN‐L specimens showed low‐grade morphology, except for a single case that showed high‐grade morphology and invasive growth. Patients with MCN‐P ranged in age from 22 to 63 years (mean age 43 years). Those with MCN‐L ranged from 43 to 69 years (mean age 57 years).

Both MCN‐P and MCN‐L expressed at least one of the specific ovarian‐like stroma markers, ER, PR, and CD10. The epithelium generally showed strong positivity for CK7 and CK19, and lower expression levels of CK20. Additionally, the epithelium component showed variable positivity for markers of pancreatic, gastric, and intestinal differentiation, and few scattered cells were found positive for synaptophysin and chromogranin A (Figure 1C and supplementary material, Table S4).

The oldest sample in the cohort, an MCN‐P sample, failed the DNA methylation quality control by having a detection *p* value higher than 0.01 for 14% of the CpG sites. Due to the poor DNA quality, it had to be excluded from the target DNA sequencing and DNA methylation analyses. Targeted DNA sequencing revealed that 9/20 samples were *KRAS* mutated and that the invasive MCN‐P sample displayed both *KRAS* and *TP53* mutations (Figure 1C).

By comparing immunohistochemical expression between MCN‐P and MCN‐L, we observed that annexin A10 (ANXA10), homeobox protein CDX2 (CDX2), mucin 1 (MUC1), and mucin 5AC (MUC5AC) were significantly higher in MCN‐P than in MCN‐L (*p* = 0.001, *p* = 0.035, *p* = 0.002, and *p* = 0.035, respectively), whereas all other markers were similarly expressed between the two entities (Figure 1D).

When comparing low‐grade lesions with high‐grade/invasive lesions of both MCN‐P and MCN‐L, we observed a significant decrease in PR expression (*p* = 0.016) and a trend towards decreased CD10 expression in high‐grade/invasive samples compared with low‐grade samples (Figure 1E). In addition, we observed that high‐grade/invasive lesions showed higher expression of CK20, CDX2, and chromogranin A compared with low‐grade lesions.

### Mucinous cystic neoplasms of the pancreas in the pancreatic context

**Figure 2:**
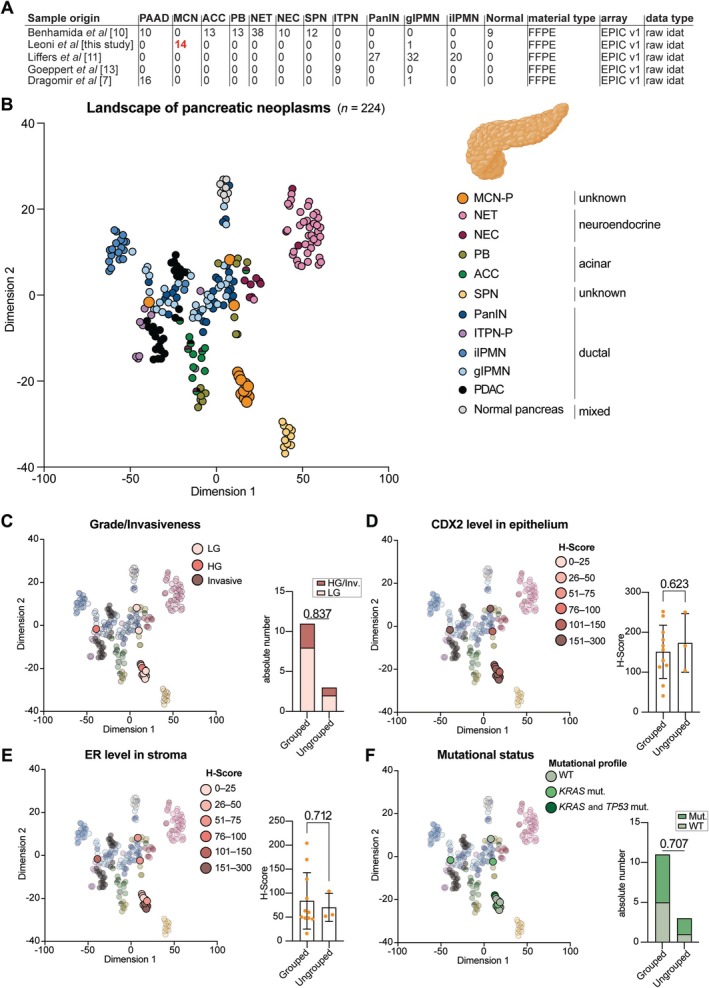
Mucinous cystic neoplasms of the pancreas in the epigenetic landscape of pancreatic neoplasms. (A) Overview of the samples composing the landscape of pancreatic entities, including the first author of the study, number and type of samples, material (FFPE or fresh frozen), array, and data type. (B) Two‐dimensional representation of the MCNs in the pancreatic context cohort (*n* = 224) using the *t*‐SNE method based on DNA methylation profiles. Colors represent histological diagnoses according to the World Health Organization classification. (C) Same *t*‐SNE, where colors represent the grade/invasiveness of the samples. Distribution of low‐ and high‐grade/invasive MCN‐P samples in the grouped and ungrouped subsets. (D) Same *t*‐SNE, where colors represent the CDX2 expression in the epithelium of MCN samples. Comparison of CDX2 expression levels between MCN‐P samples in the grouped and ungrouped subsets. (E) Same *t*‐SNE, where colors represent the ER expression level in the ovarian‐like stroma of the MCN‐P samples. Comparison of ER expression levels between MCN‐P samples in the grouped and ungrouped subsets. (F) Same *t*‐SNE, where colors represent the mutational status. Distribution of WT and *KRAS*/*KRAS* and *TP53* mutated MCN‐P samples in the grouped and ungrouped subsets. Panel B was created using BioRender.com.

Next, for MCN‐P, we constructed a comprehensive DNA methylation‐based landscape of pancreatic neoplasms by assembling one of the largest and most heterogeneous DNA methylation cohorts of pancreatic lesions. This cohort included PDAC (*n* = 26), intraductal tubulopapillary neoplasms of the pancreas (ITPN‐P, *n* = 9), pancreatic intraepithelial neoplasias (PanIN, *n* = 27), intestinal intraductal papillary mucinous neoplasms (iIPMNs, *n* = 20), gastric intraductal papillary mucinous neoplasms (gIPMNs, *n* = 33), pure and mixed acinar cell carcinomas (ACCs, *n* = 13), solid pseudopapillary neoplasms (SPNs, *n* = 12), pancreatoblastoma (PB, *n* = 13), neuroendocrine carcinoma (NEC, *n* = 10), neuroendocrine tumours (NETs, *n* = 38), and normal pancreatic tissue (*n* = 9). We added 14 MCN‐P study samples to this cohort (Figure 2A), thereby expanding the DNA methylation landscape of pancreatic neoplasms previously created by Benhamida *et al* [10].

We began by conducting a *t*‐distributed stochastic neighbor embedding (*t*‐SNE) dimensionality reduction analysis on the DNA methylation profiles. This analysis revealed several spatially related groups of pancreatic neoplasms: (1) an acinar group consisting of ACC and PB tumors; (2) an SPN group exclusively containing SPN tumors; (3) a neuroendocrine group including NET and some of the NEC samples; (4) ductal group 1 comprising PDAC, ITPN‐P, and some of the gIPMNs and PanINs; (5) ductal group 2 containing the remaining gIPMNs, PanINs, and some of the NEC and PB samples; (6) a group representing normal pancreas tissue; and (7) an MCN group containing the majority of MCN‐P samples (Figure 2B and supplementary material, Table S5).

Next, we performed unsupervised hierarchical clustering based on the DNA methylation profiles, which again demonstrated that MCN‐P formed a distinct cluster, with only a few exceptions (supplementary material, Figure S2). Overall, these data underline the fact that MCN‐P represents a distinct pancreatic entity also from an epigenetic perspective.

We observed that three MCN‐P samples were not in close proximity to the main MCN‐P group in the *t*‐SNE, suggesting a lack of local relationships, an observation further supported by the hierarchical clustering analysis. Therefore, we investigated whether factors such as grade/invasiveness, epithelial marker expression (CDX2 was selected because of the highest H‐score range among all samples), ER expression in the stroma (highest H‐score range among all samples), or mutational status could explain this divergence. Interestingly, grade/invasiveness could not explain the mixing of these three samples with the ductal groups, as their distribution was similar to that of the main MCN‐P group (*p* = 0.837, Figure 2C). Furthermore, CDX2 expression in the epithelium was similar between the main MCN‐P group and the three mismatched samples (*p* = 0.623, Figure 2D). Similarly, ER expression levels in the stroma did not significantly differ between the main MCN‐P group and the three mixed samples (*p* = 0.712, Figure 2E). The distribution of mutated samples (*KRAS* mutated and *KRAS* and *TP53* mutated) versus wild‐type (WT) samples was also similar between the main MCN‐P cluster and the three ungrouped samples (*p* = 0.707, Figure 2F).

### Mucinous cystic neoplasms of the liver in the hepatic context

**Figure 3:**
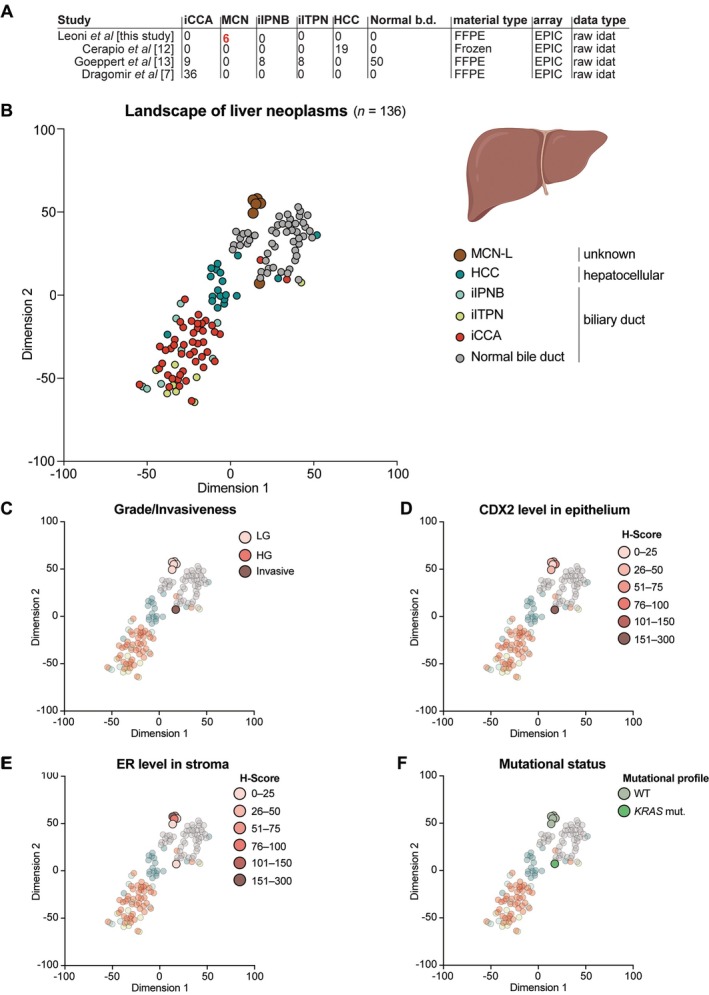
Mucinous cystic neoplasms of the liver in the epigenetic landscape of liver neoplasms. (A) Overview of the samples composing the landscape of hepatic entities, including the first author of the study, number and type of samples, material (FFPE or fresh frozen), array, and data type. (B) Two‐dimensional representation of the MCN‐L in the hepatic context cohort (*n* = 136) using the *t*‐SNE method based on DNA methylation profiles. Colors represent histological diagnoses according to the World Health Organization classification. (C) Same *t*‐SNE, where colors represent the grade/invasiveness of the samples. (D) Same *t*‐SNE, where colors represent the CDX2 expression in the epithelium of MCN samples. (E) Same *t*‐SNE, where colors represent the ER expression level in the ovarian‐like stroma of the MCN samples. (F) Same *t*‐SNE, where colors represent the mutational status. Panel B was created using BioRender.com.

Next, we examined how MCN‐L groups with neoplasms of hepato‐biliary origin. We analyzed DNA methylation data from hepatocellular carcinoma (HCC, *n* = 19), intrahepatic cholangiocarcinoma (iCCA, *n* = 45), intrahepatic intraductal papillary neoplasms of the bile duct (iIPNB, *n* = 8), intrahepatic intraductal tubulopapillary neoplasms of the bile duct (iITPN, *n* = 8), and normal bile duct tissue (*n* = 50) (Figure 3A).

In a *t*‐SNE dimensionality reduction analysis, all but one MCN‐L sample formed a distinct group, suggesting strong local relationships. Other well‐defined groups included the HCC group, the iCCA and precursor group (iIPNB and iITPN), and the normal bile duct group (Figure 3B and supplementary material, Table S5). Unsupervised hierarchical clustering analysis showed a similar distribution, with the ungrouped MCN‐L sample clustering with iCCA (supplementary material, Figure S3). Notably, this sample was the only invasive MCN‐L, showing high levels of epithelial markers and somewhat lower levels of ER in the stroma (Figure 3C–E). In addition, this was the only *KRAS*‐mutated sample (Figure 3F). These findings suggest that the more aggressive phenotype of this sample is reflected at the epigenetic level, as its DNA methylation profile more closely resembles that of iCCA rather than low‐grade MCN‐L.

### Mucinous cystic neoplasms of the pancreas and liver in the ovarian context

**Figure 4:**
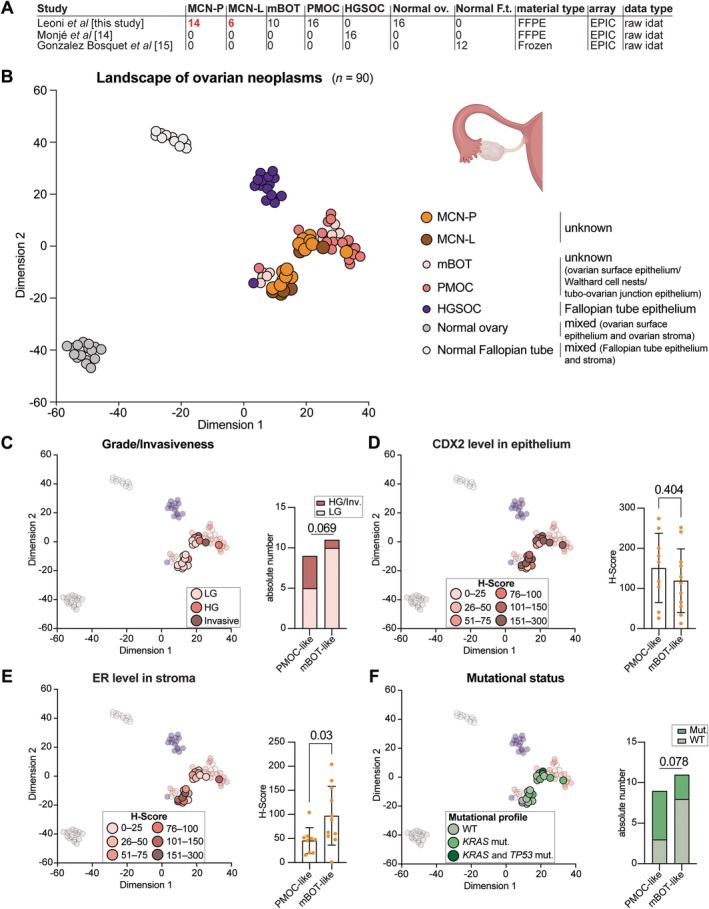
Mucinous cystic neoplasms of the pancreas and liver in the epigenetic landscape of ovarian neoplasms. (A) Overview of the samples composing the landscape of ovarian entities, including the first author of the study, number and type of samples, material (FFPE or fresh frozen), array, and data type. (B) Two‐dimensional representation of the MCNs in the ovarian context cohort (*n* = 90) using the *t*‐SNE method based on DNA methylation profiles. Colors represent histological diagnoses according to the World Health Organization classification. (C) Same *t*‐SNE, where colors represent the grade/invasiveness of the samples. Distribution of low‐grade and high‐grade and invasive MCN samples in the PMOC‐like subgroup and mBOT‐like subgroup. (D) Same *t*‐SNE, where colors represent the CDX2 expression in the epithelium of MCN samples. Comparison of CDX2 expression level between PMOC‐like and mBOT‐like MCN samples. (E) Same *t*‐SNE, where colors represent the ER expression level in the ovarian‐like stroma of the MCN samples. Comparison of ER expression level between PMOC‐like and mBOT‐like MCN samples. (F) Same *t*‐SNE, where colors represent the mutational status. Distribution of WT and *KRAS*/*KRAS* and *TP53* mutated samples in the PMOC‐like and mBOT‐like subgroups. Panel B was created using BioRender.com.

It has been proposed that MCNs may originate from remnants of the developing gonads during embryogenesis [6]. Therefore, we wanted to see how these lesions group with ovarian carcinomas, normal ovary, and normal Fallopian tube. We collected a set of primary mucinous ovarian carcinomas (PMOCs, *n* = 16), mBOTs (*n* = 10), high‐grade serous ovarian carcinomas (HGSOCs, *n* = 16), 16 normal ovaries (for which we carefully sampled both normal ovarian stroma and surface epithelium; supplementary material, Table S6), and 12 normal Fallopian tubes (Figure 4A). We observed that MCN‐P/‐L grouped together with PMOC and mBOT, while HGSOC, normal ovarian tissue, and normal Fallopian tube tissue formed distinct groups (Figure 4B and supplementary material, Table S5). This observation was further confirmed in hierarchical cluster analysis (supplementary material, Figure S4).

A more detailed analysis of the *t*‐SNE and hierarchical clustering revealed two subgroups of MCNs: one intermixed predominantly with PMOC samples (PMOC‐like) and the other closer to mBOT (mBOT‐like) (Figure 4B and supplementary material, Figure S4). A reanalysis of the five mBOT samples grouped with the PMOCs revealed that all of these samples were high‐grade mBOT, also termed intraepithelial carcinoma [17].

To investigate this, we compared the two groups. We noticed an enrichment of high‐grade and invasive samples in the PMOC‐like subgroup compared with the mBOT‐like one (*p* = 0.069, Figure 4C). Regarding the expression of an epithelial marker (CDX2), we observed no significant differences between the two groups (*p* = 0.404, Figure 4D). Consistent with our previous observations, we noticed higher ER levels in the mBOT‐like group compared with the PMOC‐like group (*p* = 0.03, Figure 4E). Furthermore, we observed an enrichment of *KRAS* mutations in the PMOC‐like group versus the mBOT‐like group (*p* = 0.078, Figure 4F).

These findings indicate that MCN samples not only grouped with mucinous ovarian tumors but also showed a distribution pattern along the progression from low‐grade to high‐grade/invasive, similar to that of mBOT and PMOC, suggesting further DNA‐methylation profile similarities across these tumor types.

### Mucinous cystic neoplasms of the pancreas and liver in the hepato‐pancreato‐ovarian context

**Figure 5:**
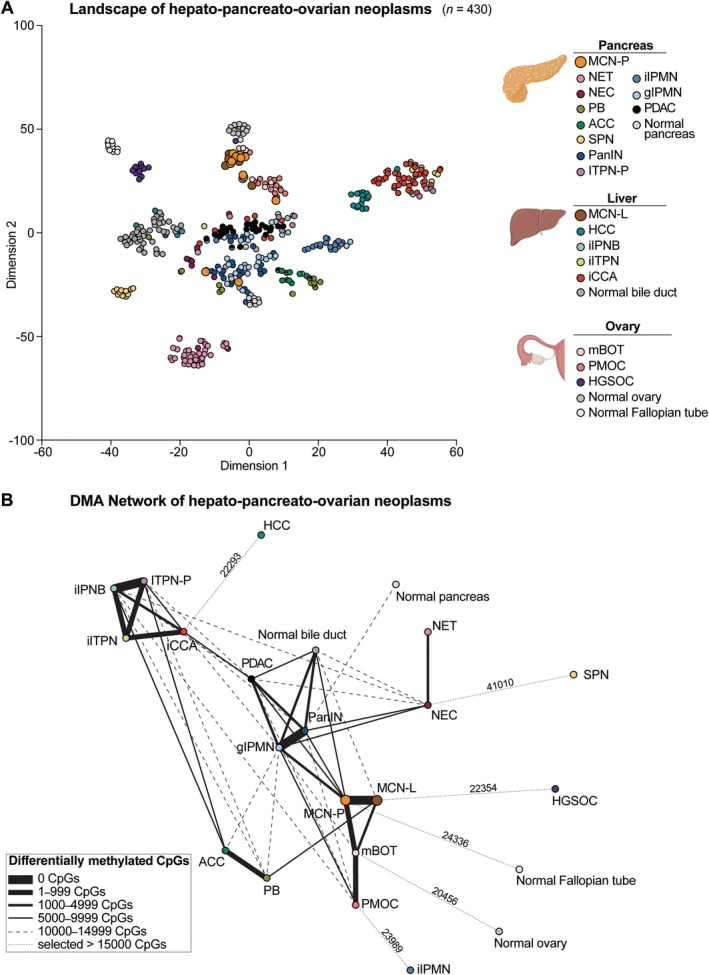
Relatedness of MCNs in the hepato‐pancreato‐ovarian neoplasm landscape. (A) *t*‐SNE of the hepato‐pancreato‐ovarian neoplasms. Different tumor entities according to the World Health Organization classification are marked with different colors. Each dot represents a unique patient. (B) Relatedness network of the hepato‐pancreato‐ovarian neoplasms. Different tumor entities according to the World Health Organization classification are marked with different colors. Each dot represents all patients with a specific tumor entity; the edges represent the number of differentially methylated CpGs between the 23 different entities. DMA, differential methylation analysis. Panel A was created using BioRender.com.

Next, we performed a *t*‐SNE analysis including all the pancreatic, hepatic, and ovarian neoplasms analyzed above, encompassing 23 different entities and 430 unique patients. We observed the same distinct groups as previously described with only a few differences (Figure 5A and supplementary material, Figure S5A). Focusing on MCN‐P/‐L, we noticed the grouping with mBOT and PMOC, and also with normal ovary, exhibiting a similar distribution along the mBOT–PMOC progression line. Two of the three MCN‐P samples that initially grouped towards pancreatic ductal lesions were again mixed with lesions of ductal origin (Figure 5A). Curiously, along with these two MCN‐P samples, one PMOC sample also moved towards ductal lesions (Figure 5A). To have a quantitative perspective on the relationship between the DNA methylation profiles of the samples, we performed agglomerative consensus clustering on the same 10,000 CpGs used for heatmap and *t*‐SNE analysis. As expected, MCN‐P/‐L as well as mBOT, PMOC, and normal ovary clustered together (supplementary material, Figure S5B,C), showing a high degree of consensus. Indeed, a statistical comparison of the degree of consensus within MCN‐P/‐L samples versus all other groups revealed non‐significant differences only versus mBOT, normal ovary, and MCN‐L/‐P, respectively (supplementary material, Figure S5D,E).

To better understand the relationship between these entities, we performed a network analysis where each node represents all samples with a given entity (23 entities), and each edge represents the number of differentially methylated CpGs between the different entities (the fewer differentially methylated CpGs, the stronger the association between two entities; supplementary material, Table S7). We observed some well‐known close relationships from classical morphology studies or genetic profiling; for example, the closest entity to NET was NEC, and as expected, ACC was closely related to PB. Some entities remained isolated because no related lesions were included in the analysis, such as HCC and HGSOC, or due to their very specific molecular profiles, such as SPN (Figure 5B).

Regarding MCN‐P and MCN‐L, we noticed a very close relationship between the two, with zero differentially methylated CpGs between them. For both MCN‐P and MCN‐L, the most closely related neoplasm was mBOT, further strengthening our observation that MCN and ovarian mucinous neoplasms share a similar DNA methylation profile. In addition, the network analysis revealed other interesting relationships: MCN‐P shares a high degree of similarity with gIPMN and some similarities with PDAC and PanIN (Figure 5B), possibly explaining the two MCN‐P samples that clustered with ductal lesions. This could also be confirmed at the IHC level, where we observed higher levels of MUC1 and MUC5AC in MCN‐P than in MCN‐L (Figure 1D).

### Pathway analysis and cellular decomposition of the epigenetic profile of mucinous cystic neoplasms of the pancreas and liver

**Figure 6:**
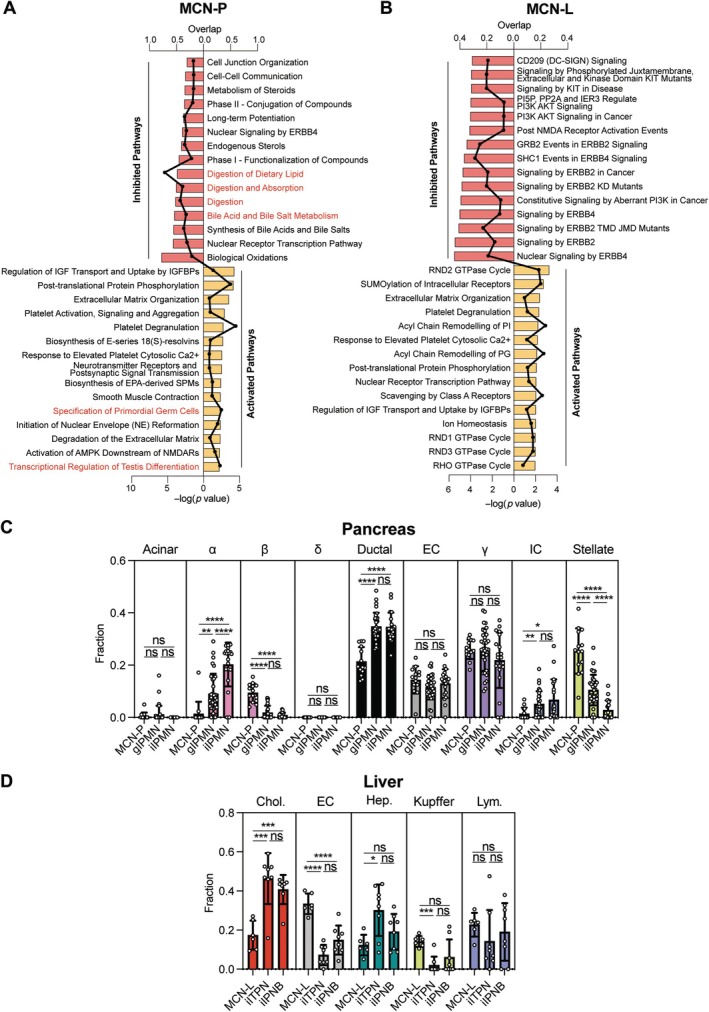
Pathway analysis and cellular decomposition of the epigenetic profile of mucinous cystic neoplasms of the pancreas and liver. (A) Dysregulated pathways (Reactome) for the genes associated with the differentially methylated transcription start sites in MCN‐P versus normal pancreas. (B) Dysregulated pathways (Reactome) for the genes associated with the differentially methylated transcription start sites in MCN‐L versus normal bile duct. (C) Estimated cell‐type fraction for MCN‐P, gIPMN, and iIPMN using as reference normal pancreas specific single‐cell RNA‐sequencing. Acinar, acinar cell; α, endocrine alpha cell; β, endocrine beta cell; δ, endocrine delta cell; Ductal, ductal cell; EC, endothelial cell; γ, endocrine gamma cell; IC, immune cell; Stellate, stellate cell. (D) Estimated cell‐type fraction for MCN‐L, iITPN, and iIPNB using as reference normal liver specific single‐cell RNA‐sequencing. Chol., cholangiocyte; EC, endothelial cell; Hep., hepatocyte; Kupffer, Kupffer cell; Lym., lymphocyte; ns, not significant. *p < 0.05; **p < 0.01; ***p < 0.001; ****p < 0.0001.

To gain a more specific understanding of MCN molecular alterations, we performed pathway analysis. First, we extracted differentially methylated CpGs between MCN‐P and normal pancreas and MCN‐L and normal bile duct tissue; selected those located at transcription start sites (TSSs); and mapped them to corresponding genes. We considered genes corresponding to hypermethylated CpGs in TSSs to be inhibited in MCN, and those corresponding to hypomethylated CpGs in TSSs to be activated in MCN. For MCN‐P versus normal pancreas, we observed, among others, an activation of pathways related to primordial germ cells and their differentiation, and an inhibition of pathways related to digestion (Figure 6A). For MCN‐L versus normal bile duct, we observed activation of non‐specific signal transduction pathways and inhibition of pathways related to ERBB2 and ERBB4 signaling (Figure 6B). We attributed the inhibition of ERBB signaling to a decrease in immune cells [18] in MCN‐L compared with normal bile duct. Therefore, second, we deconvoluted the corresponding transcriptome data using mid‐gestation whole‐body single‐cell RNA sequencing data from the DESCARTES database [19]. For MCN‐P compared with normal pancreas, we observed an enrichment of stromal and vascular endothelial cells and a depletion of cells with a role in digestion (acinar cells and ductal cells in pancreas, among others; supplementary material, Figure S6A). Similarly, in MCN‐L compared with normal bile duct, we observed an enrichment of stromal and vascular endothelial cells and, as hypothesized, a depletion of immune cells (supplementary material, Figure S6B).

Finally, to gain a more granular understanding of cellularity, we directly decomposed the methylation profile of MCN‐P and MCN‐L on adult tissue‐specific single‐cell RNA sequencing data (normal pancreas and normal liver, respectively) without using matched gene expression as a proxy [20]. To this end, we compared MCN lesions with cystic and intraductal precursor lesions of the pancreas (gIPMN and iIPMN) and bile duct (iITPN and iIPNB). MCN‐P showed significantly lower fractions of alpha and ductal cells and significantly higher levels of stellate and beta cells compared with gIPMN and iIPMN (Figure 6C). MCN‐L decomposition on liver tissue showed significantly lower levels of cholangiocytes and hepatocytes (suggesting low contamination of our samples) and significantly higher levels of endothelial cells and Kupffer cells compared with iITPN and iIPNB (Figure 6D). Collectively, these data reinforce that MCNs are distinct lesions of the hepato‐pancreato‐biliary system, characterized particularly by their stromal component, and suggest a possible primordial germ cell origin.

## Discussion

The true origin of MCN‐P and MCN‐L remains a subject of considerable research interest. Herein, we demonstrate that MCN‐P and MCN‐L are distinct entities within the landscape of pancreatic and hepatic tumors, also from an epigenetic perspective. Additionally, our data confirm that MCNs are closely related to mucinous ovarian tumors. We demonstrated this by performing a broad immunohistochemical characterization, targeted DNA sequencing, and array‐based DNA methylation analysis.

The immunohistochemical analysis revealed several differences between MCN‐P and MCN‐L. First, we found that the epithelium of MCN‐P had a significantly higher level of annexin A10 compared with that of MCN‐L. Annexin A10 has been proposed as a useful marker in differentiating PDAC liver metastases from iCCA [7, 21]. On the other hand, our data show that PMOC samples are always negative for annexin A10 (data not shown). This observation potentially suggests that the epithelial component of MCN lesions may be of pancreatic/biliary origin and not derived from primordial germ cells. This observation is consistent with the pathogenic mechanism proposed by Kumata *et al*, who suggested that the ovarian‐like stroma of MCNs plays a critical role in their pathogenesis. The authors suggested that androgens locally produced by the ovarian‐like stroma induce malignant transformation of ductal or biliary epithelium in a paracrine manner [22]. In another study analyzing the pathogenesis of MCN‐P, it was shown that the ovarian‐like stroma characteristic of MCN‐P exhibits marked overexpression of certain genes, including steroidogenic acute regulatory protein, estrogen receptor 1, and secreted frizzled‐related protein, a key modulator of Wnt signaling. This genetic profile is notably different from the fibrous stroma observed in chronic pancreatitis, suggesting a unique stromal environment in MCN‐P [23]. Consistent with this hypothesis, Sano *et al* developed the first mouse model that develops MCN‐P. By overexpressing Wnt1 in the pancreatic acinar cells of female *LSL‐Kras G12D, Ptf1a‐cre* mice, the authors induced the development of cystic lesions with ovarian‐like stroma. The same activation of Wnt signaling within the stromal cells (β‐catenin nuclear expression) was also found in human MCN‐P samples, but not in PanIN or PDAC [24]. Taken together, these data suggest that the ovarian‐like stroma may represent the *primum movens* in the pathogenesis of MCN, with the epithelial component likely arising from a local origin.

Second, we observed that CDX2, MUC1, and MUC5AC are more highly expressed in the epithelium of MCN‐P than in that of MCN‐L. This further potentially supports the concept of a local origin of the epithelial components of MCNs and suggests a possible intestinal, pancreatic, or gastric differentiation of the epithelium. Further mechanistic studies are needed to confirm this hypothesis.

Third, regarding the stromal component, we observed a decrease in CD10, and especially PR from low‐grade to high‐grade/invasive lesions, which may represent a progressive involution of the ovarian‐like stroma. This observation is not perfectly aligned with the data of Fukumura *et al*, who demonstrated a significant correlation of tumor size with patient age, histological grade, and ovarian‐like stroma with intralobular distribution, but not with the amount of ovarian‐like stroma [25].

Our targeted DNA sequencing was consistent with the existing literature, revealing an increase in *KRAS* mutations from low‐ to high‐grade lesions and the addition of *TP53* mutations for the invasive tumors [3, 26]. The *RNF43* mutation described in MCN‐P [3] was unfortunately not covered by our DNA targeted sequencing panel.

Next, DNA methylation profiles of MCN‐P in the pancreatic landscape grouped together and were distinct from other entities such as IPMN and PanIN. As discussed by Benhamida *et al*, DNA methylation analysis can help group pancreatic tumors based on their cell of origin, leading to a better diagnosis [10]. Compared with Benhamida *et al*, we have expanded the DNA methylation landscape of pancreatic neoplasms by adding gIPMN, iIPMN, NEC, and MCN‐P, as well as a larger number of PDAC samples. The clinical utility of such landscapes to develop DNA methylation classifiers for PDAC metastases or solid pancreatic neoplasms has recently been demonstrated [27, 28], and we consider that with the additional data presented here, the DNA methylation classifiers can be expanded to cystic lesions, such as IPMN and MCN. Furthermore, we noticed that besides the main group of MCN‐P, three MCN‐P samples were separately grouped with pancreatic ductal lesions. Curiously, neither the expression of the stromal and epithelial marker with the widest distribution among the samples, nor the mutational status, nor the grading could explain this.

In the hepatic context, we observed that all MCN‐L samples grouped separately from all other hepatic lesions, except for one sample that was located close to normal bile duct samples. This sample presented high‐grade epithelial atypia and an epigenetic profile more similar to iCCA than to MCN‐L. Moreover, detailed analysis revealed that this sample also harbored a *KRAS* mutation.

Furthermore, we show for the first time that MCN‐P and MCN‐L are, from an epigenetic standpoint, very similar to mucinous ovarian tumors. Strengthening this finding, we noticed that low‐grade MCN co‐localized predominantly with mBOT, whereas high‐grade/invasive MCN generally grouped with PMOC. This observation is consistent with the observation that mBOT and PMOC are genomically similar to pancreatic tumors [4]. In addition, we show that MCNs are even more similar to mBOT/PMOC than to PDAC samples. Consistent with our findings, Elias *et al* postulated a common origin between MCN‐P and mBOT/PMOC using array‐based gene expression sequencing. They demonstrated that MCN‐P grouped together with mucinous ovarian tumors while remaining distinctly separate from PDAC. Furthermore, their results indicated that both mucinous ovarian tumors and MCN‐P grouped near primordial germ cells, suggesting a common cell of origin [6]. We confirmed this analysis from an epigenetic standpoint and extended it to include not only MCN‐P but also MCN‐L, along with other pancreatic and hepatic lesions.

Fourth, we analyzed all samples together to create a landscape of hepato‐pancreato‐ovarian neoplasms. We employed two approaches: dimensionality reduction (*t*‐SNE) and clustering based on the CpGs with the highest standard deviation, and network analysis based on DMPs. The *t*‐SNE revealed that most MCN‐P and all MCN‐L grouped together with mBOT, PMOC, and normal ovary. Again, two of the three MCN‐P samples that were separated from the main group in the pancreatic landscape grouped together with PDAC and its precursors. Interestingly, these samples were followed by one PMOC, further strengthening the similarities between mucinous ovarian tumors and PDAC observed at the genomic level [4]. Agglomerative consensus clustering confirmed these observations at a quantitative level, showing significantly different degrees of consensus between MCN‐P/MCN‐L and all other tissue types with the exception of mBOT, normal ovary, and MCN‐L/‐P, respectively, which showed non‐significant differences, and less significant differences with PMOC. Similar to our previous work [29], we used network theory to further decipher this scenario and built a network where the edges represent DMPs and the nodes represent all samples with the same diagnosis. This completely different approach showed that MCN‐P and MCN‐L are very similar to each other and that their closest entity is mBOT. In addition, we observed some degree of relatedness between MCN‐P and gIPMN, PanIN, and PDAC, potentially explaining the higher levels of some epithelial markers and the grouping of the three MCN‐P samples with pancreatic ductal lesions.

Finally, exploratory mechanistic analysis suggested an activation of germ cell differentiation pathways in MCN‐P and an enrichment of stromal cells such as stellate cells in MCN‐P, further strengthening the role of the stroma, also at the molecular level, in the characterization of these cystic lesions.

Our study has several limitations that we would like to outline. First, the entire construct is based on accurate diagnosis. For the in‐house MCN samples, we were very stringent regarding the diagnosis. The diagnosis of MCN is theoretically straightforward. Nevertheless, we also performed DNA methylation analysis on a gIPMN sample (82‐year‐old female) that we initially thought was MCN‐P. The lesion presented as multicystic with a foveolar‐like prismatic and partially flattened epithelium, and a relatively fibrous stroma in which sparse groups of cells were positive for PR. These groups of cells were also positive for synaptophysin and chromogranin A, suggesting that they were entrapped Langerhans islet cells. This highlights a pitfall that we identified during the collection and immunohistochemical analysis of the cases: PR‐positive Langerhans islet cells entrapped in the fibrous wall can be confused with ovarian‐like stroma. These cells have a more organoid distribution and round nuclei compared with true ovarian‐like stroma, which is more dispersed and shows elongated nuclei. The DNA methylation analysis showed that this sample clustered with the other gIPMNs. The reclassification of this sample clearly demonstrates the clinical utility of DNA methylation for the diagnosis of pancreatic cystic lesions. Second, we performed macrodissection of the lesions, but we consider that a compartmentalized microdissection is necessary for a more granular analysis. Indeed, immunohistochemical studies have revealed a loss of SMAD4 expression only in the invasive epithelial compartment of MCN‐P, while the ovarian‐like stroma retained SMAD4 expression, suggesting molecular differences [30]. In addition, a recent study on MCN‐P revealed *KRAS* mutation heterogeneity in microdissected epithelial compartments, with higher proportions of *KRAS* mutations in regions with papillary architecture, columnar cells, and nuclear pseudostratification [31]. In future work, we plan to perform microdissection of the samples, analyze the stroma and different epithelial regions separately, and compare them with the epithelium and stroma of mucinous ovarian tumors. Currently, such an approach is limited by the large amount of DNA required for DNA methylation analysis.

In conclusion, we have shown that MCN‐P and MCN‐L are a distinct subtype of pancreatic and hepatic lesions and that these tumors share a similar DNA methylation profile with mucinous ovarian tumors, suggesting a possible common origin.

## Author contributions statement

MPD was responsible for the study conceptualization and design. ZL, TJ, ETT, CCMN, EIB, JS, TM, WS, JP, DSK, JKB and IE acquired the clinical and pathological data. ZL, EG, BC, DC, DH, SS and MPD interpreted the data. ZL, TGC, TJ, MM, SS and MPD analyzed the data. ZL and MPD drafted the manuscript. TGC and MM performed the bioinformatics analysis. MPD acquired the funding. All authors have read and approved the final paper.

## Supporting information


Supplementary materials and methods
**Figure S1.** Representative images of both MCN‐P and MCN‐L samples included in the study showing progesterone receptor (PR) positivity in the ovarian‐like stroma
**Figure S2.** Hierarchical clustering of the samples composing the landscape of pancreatic neoplasms
**Figure S3.** Hierarchical clustering of the samples composing the landscape of liver neoplasms
**Figure S4.** Hierarchical clustering of the samples composing the landscape of ovarian neoplasms
**Figure S5.** Hierarchical and consensus clustering and sample clustering of hepato‐pancreato‐ovarian neoplasms
**Figure S6.** Cell type enrichment analysis for genes linked to differential TSS methylation in MCN‐P and MCN‐L tissues
**Table S1.** Characteristics of the antibodies used in immunohistochemical (IHC) analysis
**Table S2.** Parameters used for quantitative expression analysis using QuPath version 0.5.1 (Queen's University, Belfast, Northern Ireland) for epithelial and stromal immunohistochemistry markers
**Table S3.** Clinical and pathological characteristics and the DNA methylation array scan ID of the included samples
**Table S4.** H‐scores of epithelial and stromal markers quantified using QuPath version 0.5.1 (Queen's University, Belfast, Northern Ireland)
**Table S5.** Top standard deviated CpGs used for building the landscape of pancreatic, hepatic, ovarian, and hepato‐pancreato‐ovarian neoplasms (Excel file)
**Table S6.** Normal ovary cases: type of surgery, purpose of surgery, and histological description of the ovarian tissue
**Table S7.** Number of differentially methylated CpGs with an adjusted *p* value less than 0.01 and an absolute log FC value greater than 0.3 between the different entities (Excel file)

## Data Availability

DNA methylation array data are available from the Gene Expression Omnibus (GEO) repository under the following accession number: GSE293725.
