# Supplementary material for: Mucinous cystic neoplasms of the pancreas and liver share a similar DNA methylation profile with mucinous ovarian tumors
Source: J Pathol. 2025 Jun 25;267(1):10–24. doi: 10.1002/path.6439 (PMC12337810; doi:10.1002/path.6439)
Supplement: Supplementary file 1 — Supplementary materials and methods Figure S1. Representative images of both MCN‐P and MCN‐L samples included in the study showing progesterone receptor (PR) positivity in the ovarian‐like stroma Figure S2. Hierarchical clustering of the samples composing the landscape of pancreatic neoplasms Figure S3. Hierarchical clustering of the samples composing the landscape of liver neoplasms Figure S4. Hierarchical clustering of the samples composing the landscape of ovarian neoplasms Figure S5. Hierarchical and consensus clustering and sample clustering of hepato‐pancreato‐ovarian neoplasms Figure S6. Cell type enrichment analysis for genes linked to differential TSS methylation in MCN‐P and MCN‐L tissues Table S1. Characteristics of the antibodies used in immunohistochemical (IHC) analysis Table S2. Parameters used for quantitative expression analysis using QuPath version 0.5.1 (Queen's University, Belfast, Northern Ireland) for epithelial and stromal immunohistochemistry markers Table S3. Clinical and pathological characteristics and the DNA methylation array scan ID of the included samples Table S4. H‐scores of epithelial and stromal markers quantified using QuPath version 0.5.1 (Queen's University, Belfast, Northern Ireland) Table S5. Top standard deviated CpGs used for building the landscape of pancreatic, hepatic, ovarian, and hepato‐pancreato‐ovarian neoplasms (Excel file) Table S6. Normal ovary cases: type of surgery, purpose of surgery, and histological description of the ovarian tissue Table S7. Number of differentially methylated CpGs with an adjusted p value less than 0.01 and an absolute log FC value greater than 0.3 between the different entities (Excel file) [file PATH-267-10-s001.zip › path6439-sup-0001-SuppMatMethFiguresS1-S6TablesS1-S4S6.docx]

**Mucinous cystic neoplasms of the pancreas and liver share a similar DNA methylation profile with mucinous ovarian tumors**

Z Leoni *et al. J Pathol* <https://doi.org/10.1002/path.6439>

**Supplementary materials and methods**

**Supplementary Figures S1–S6**

**Supplementary Tables S1–S4 and S6**

**(Supplementary Tables S5 and S7 are provided in separate Excel files)**

Reference numbers refer to the main text list

**Supplementary materials and methods**

*Immunohistochemistry*

Immunohistochemical staining was performed using a BenchMark XT immunostainer (Ventana Medical Systems, Tucson, AZ, USA). For antigen retrieval, sections were incubated in CC1 mild buffer (Ventana Medical Systems) for 30 min at 100 °C or in protease 1 for 8 min. The sections were stained with anti-cytokeratin 7 (CK7; M7018), anti-cytokeratin 19 (CK19; MU246-UC), anti-cytokeratin 20 (CK20; M7019), anti-caudal type homeobox 2 (CDX2; RBK019-05), anti-carbohydrate antigen 19-9 (CA19-9; NCL-L-CA19-9), anti-annexin A10 (ANXA10; ab213656), anti-mucin 1 (MUC1; M0613), anti-mucin 5AC (MUC5AC; 18-2322), anti-chromogranin A (chromogranin A; AC-0037), anti-synaptophysin (synaptophysin; NCL-L-SYNAP-299), anti-estrogen receptor (ER; 790-4325), anti-progesterone receptor (PR; 790-4296), and anti-CD10 (CD10; NCL-L-CD10-270) for 60 min at room temperature and visualized using the avidin–biotin complex method and DAB (3,3′-diaminobenzidine; Ventana Medical Systems) (supplementary material, Table S1). Cell nuclei were stained by additional incubation with hematoxylin and bluing reagent for 12 min (Ventana Medical Systems).

All slides stained for the above markers were digitized at 20.0 magnification (0.2425-pixel width and 0.2426-pixel height) using a 3DHISTECH P150 digital slide scanner (3DHISTECH, Budapest, Hungary). Analysis of IHC staining was performed using QuPath version 0.5.1 (downloaded from https://qupath.github.io/; Queen’s University, Belfast, Northern Ireland). Whole slide images were evaluated, and representative areas free from artefacts were selected for further quantitative analysis. The analysis was then optimized by applying different cell detection parameters for the epithelium and for the stroma. For epithelial analysis in all samples, the following parameters were applied: optical density sum detection image, pixel size 0.2 µm, intensity threshold 0.2, nucleus parameters with background radius 8 µm, minimum area 10 µm^2^, maximum area 400 µm^2^, sigma 1.5. In order to perform a semi-quantitative analysis, three intensity threshold parameters were used: 0.2 (1+), 0.4 (2+), and 0.6 (3+). The following parameters were set for stromal analysis: optical density sum detection image, pixel size 0.2 µm, intensity threshold 0.2, nucleus parameters with background radius 0 µm, minimum area 10 µm^2^, maximum area 400 µm^2^, sigma 1.5. Three intensity threshold parameters were used: 0.1 (1+), 0.2 (2+), and 0.4 (3+). The intensity of staining in the cells was then categorized into four levels: 0 (no positivity), 1+ (weak positivity), 2+ (moderate positivity), and 3+ (strong positivity) (supplementary material, Table S2). For each category, the percentage of cells exhibiting that particular staining intensity was determined using QuPath (downloaded from https://qupath.github.io/; Queen’s University, Belfast, Northern Ireland). Then we applied the formula H-score = [1 × % of cells with weak positivity (1+)] + [2 × % of cells with moderate positivity (2+)] + [3 × % of cells with strong positivity (3+)]. The H-score ranged from 0 to 300, with a score of 0 indicating no marker expression and a score of 300 indicating strong staining in all cells [14].

*DNA extraction*

To extract DNA, a new H&E slide was prepared, stained, and the area of the cystic neoplasm – including both the epithelium and the ovarian-like stroma – was identified and marked under a light microscope (Olympus BX46, Tokyo, Japan). Depending on the tumor size, between 5 and 20 FFPE 5-µm-thick slides were cut and the tumor regions were carefully macrodissected for DNA extraction. Next, semi-automated DNA extraction was performed following the manufacturer’s protocol (Maxwell RSC FFPE Plus DNA Purification Kit, Custom; Promega, Madison, WI, USA). DNA quantities were measured using the Qubit HS DNA assay (Thermo Fisher Scientific, Waltham, MA, USA). For 17 of the samples, the recommended 500 ng of DNA for DNA methylation analysis was obtained, while the additional four samples yielded as little as 210 ng of DNA.

*DNA methylation analysis and array processing*

DNA restoration was performed using the Infinium HD FFPE DNA Restore Kit (Illumina, San Diego, CA, USA) and methylation analysis was performed using the Illumina Infinium MethylationEPIC v1 BeadChip (Illumina) following the manufacturer’s protocols.

Various R packages as implemented in ChAMP [16] were used for the DNA methylation analysis. Raw signals from all the IDAT files were loaded using the minfi package. The quality control steps implemented in the ChAMP data processing pipeline were used to assess the data quality, excluding (1) probes that have fewer than three beads in at least 5% of the samples; (2) all single nucleotide polymorphism (SNP)-associated sites; (3) multi-hit sites; (4) CpGs found on chromosomes X and Y; and (5) probes with a detection *p* value greater than 0.01. Moreover, samples which had more than 10% of the probes failing the detection *p* value test were excluded from the analysis. In the last processing step, beta values were obtained and normalized using FunNorm first, followed by BMIQ.

*Targeted next-generation sequencing*

For DNA sequencing, libraries were generated using AmpliSeq for Illumina Cancer Hotspot Panel v2 (Illumina) following the manufacturer’s protocol. In brief, 80 ng of DNA from each sample was input. The cancer hotspot panel included primers for the amplification of the hotspot regions of the following genes: *ABL1*, *EGFR*, *GNAS*, *KRAS*, *PTPN11*, *AKT1*, *ERBB2*, *GNAQ*, *MET*, *RB1*, *ALK*, *ERBB4*, *HNF1A*, *MLH1*, *RET*, *APC*, *EZH2*, *HRAS*, *MPL*, *SMAD4*, *ATM*, *FBXW7*, *IDH1*, *NOTCH1*, *SMARCB1*, *BRAF*, *FGFR1*, *JAK2*, *NPM1*, *SMO*, *CDH1*, *FGFR2*, *JAK3*, *NRAS*, *SRC*, *CDKN2A*, *FGFR3*, *IDH2*, *PDGFRA*, *STK11*, *CSF1R*, *FLT3*, *KDR*, *PIK3CA*, *TP53*, *CTNNB1*, *GNA11*, *KIT*, *PTEN*, and *VHL.* The target regions were then amplified by PCR (Biometra TOne, Analytik Jena, Jena, Germany) following the manufacturer’s protocol. Next-generation sequencing (NGS) was performed using an iSeq 100 Sequencing System (Illumina).

Mutation calling was conducted using the SEQUENCE Pilot Software version 5.4.0 (JSI Medical Systems GmbH, Ettenheim, Germany). Sequencing reads were aligned to the human reference genome (hg19), and variant calling was performed using the software’s integrated pipelines. Variants were filtered to include only those with a minimum allele frequency greater than 5%. Mutations classified as pathogenic or potentially pathogenic based on established databases were selected for further analysis.

t*-distributed stochastic neighbor embedding* (t*-SNE*)

In order to create the t-distributed stochastic neighbor embedding (*t*-SNE) plots, the beta values of the top 10,000 CpG sites with the highest standard deviation in their methylation level across the samples of a given plot were selected and processed using the R package Rtnse [32] with 5,000 iterations. The perplexity (p) was selected individually for each plot in order to account for the different numbers of samples. In brief, four plots were created: (1) landscape of pancreatic neoplasms (p = 12); (2) landscape of hepatic neoplasms (p = 10); (3) landscape of ovarian neoplasms (p = 7); and (4) landscape of hepato-pancreato-ovarian neoplasms (p = 17).

*Unsupervised clustering: heatmap and agglomerative consensus*

The heatmaps were created using the R package ComplexHeatmap [33] on the same 10,000 CpG sites used for building the *t*-SNEs. The order of the samples in the heatmap is given by a hierarchical clustering model that is automatically run by the R package.

The agglomerative consensus similarity matrix and box plots were computed using the consensus clustering method [34], implemented in the Python library consensus clustering, with the agglomerative clustering algorithm, as implemented in the Python package sklearn [35]. The models were fitted with the same samples and the top 10,000 most standard deviated CpGs used for plotting the *t*-SNE of the landscape of the hepato-pancreato-ovarian neoplasms. The beta values of the CpGs were decomposed into principal components using the PCA (principal component analysis) method in sklearn before being used in the clustering model. The consensus clustering was conducted for a number of 13 clusters, with 200 resamples, and a resample fraction of 0.7.

*Pathway and cell type analysis*

For the pathway and cell type analysis, we started from the differentially methylated probes (DMPs) between MCN-P and normal pancreas tissue and MCN-L and normal bile duct tissue. The same criteria for DMPs used for constructing the differentially methylated networks were used for pathway analysis (adjusted *p* value < 0.01 and an absolute log FC value > 0.3). We next selected only the DMP located in the transcription start site (TSS) (both TSS200 and TSS1500) and matched the probes to the corresponding genes. We considered genes matching hypomethylated probes in MCN versus normal as activated and those matching hypermethylated probes in MCN versus normal as inhibited. Integrated pathway analyses were performed with Reactome Pathways 2024 using Enrichr bioinformatics resources (http://amp.pharm.mssm.edu/Enrichr/, last accessed 15 March 2025). *p*< 0.01 was considered significant. Results were presented as bar charts.

Cell type deconvolution was performed with Descartes Cell Types and Tissue 2021 using Enrichr bioinformatics resources (http://amp.pharm.mssm.edu/Enrichr/, last accessed 15 March 2025). *p*< 0.01 was considered significant. Results were visualized using bar charts.

*EpiSCORE*

Cell deconvolution was performed using the R library EpiSCORE [36]. The proportion of cell types present in a mixed cell population is estimated based on reference single-cell RNA-seq data. The algorithm uses a non-negative least squares regression to output said cell proportion values.

The preprocessed beta values of all CpGs were compared with reference DNA methylation signature matrices provided by the authors of EpiSCORE. The MCN-P, iIPMN, and gIPMN samples were analyzed using pancreas reference DNA methylation signature matrices, while MCN-L, iITPN, and iIPNB samples were analyzed using liver reference signatures. All other EpiSCORE parameters were kept at default values.

**
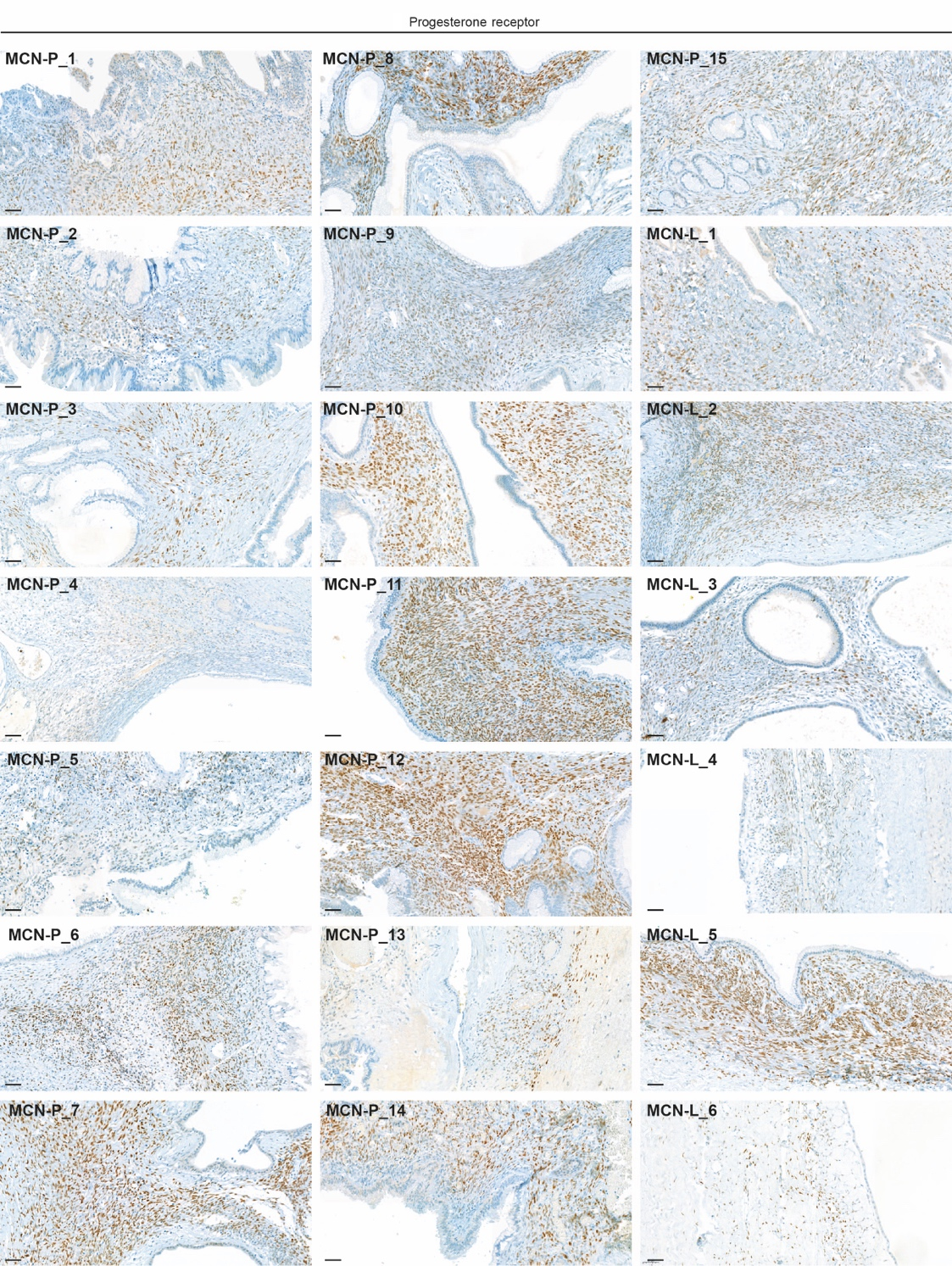
**

**Figure S1.** Representative images of both MCN-P and MCN-L samples included in the study showing progesterone receptor (PR) positivity in the ovarian-like stroma. Scale bar length: 50 μm.

**
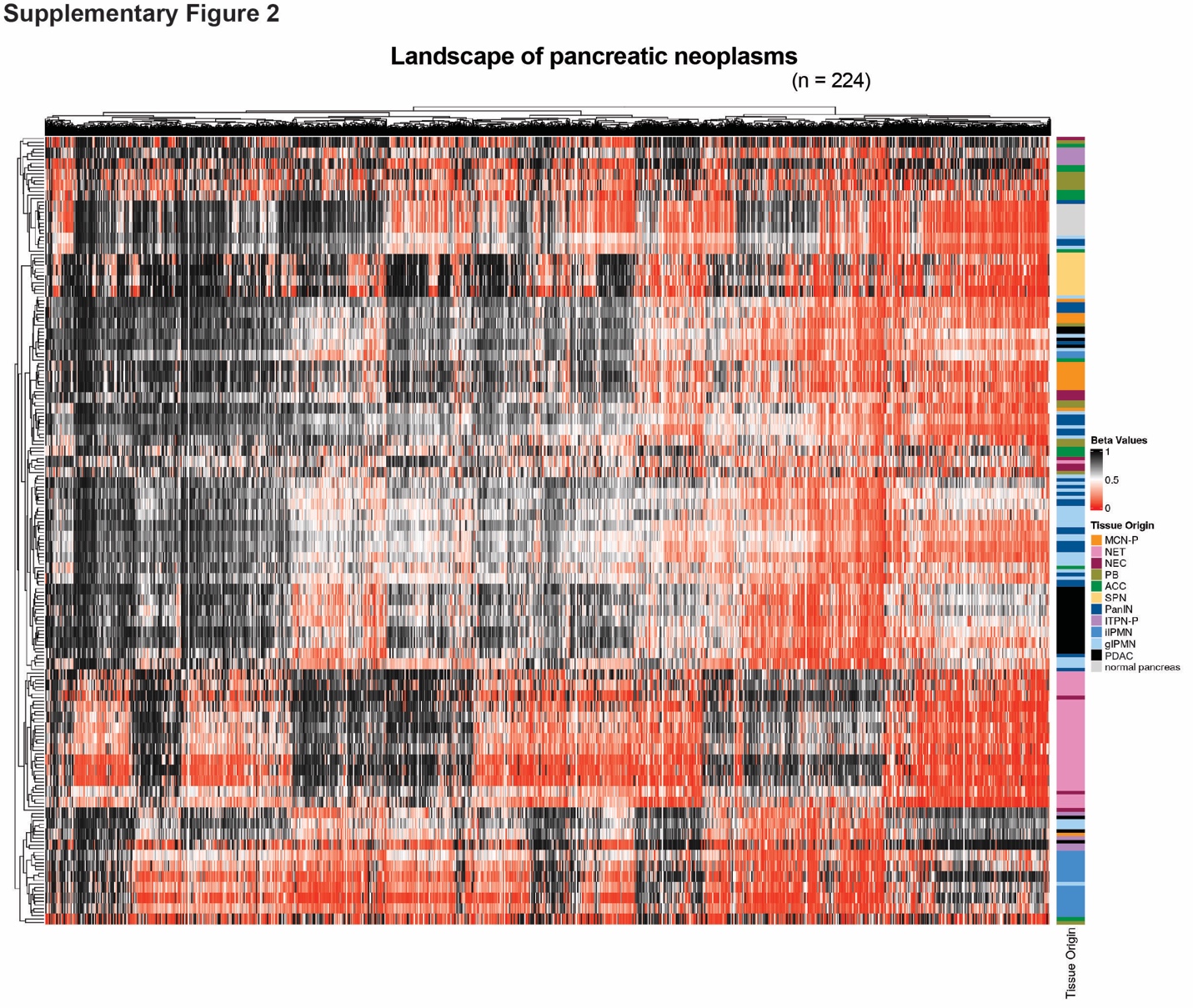
**

**Figure S2.** Hierarchical clustering of the samples composing the landscape of pancreatic neoplasms.

**
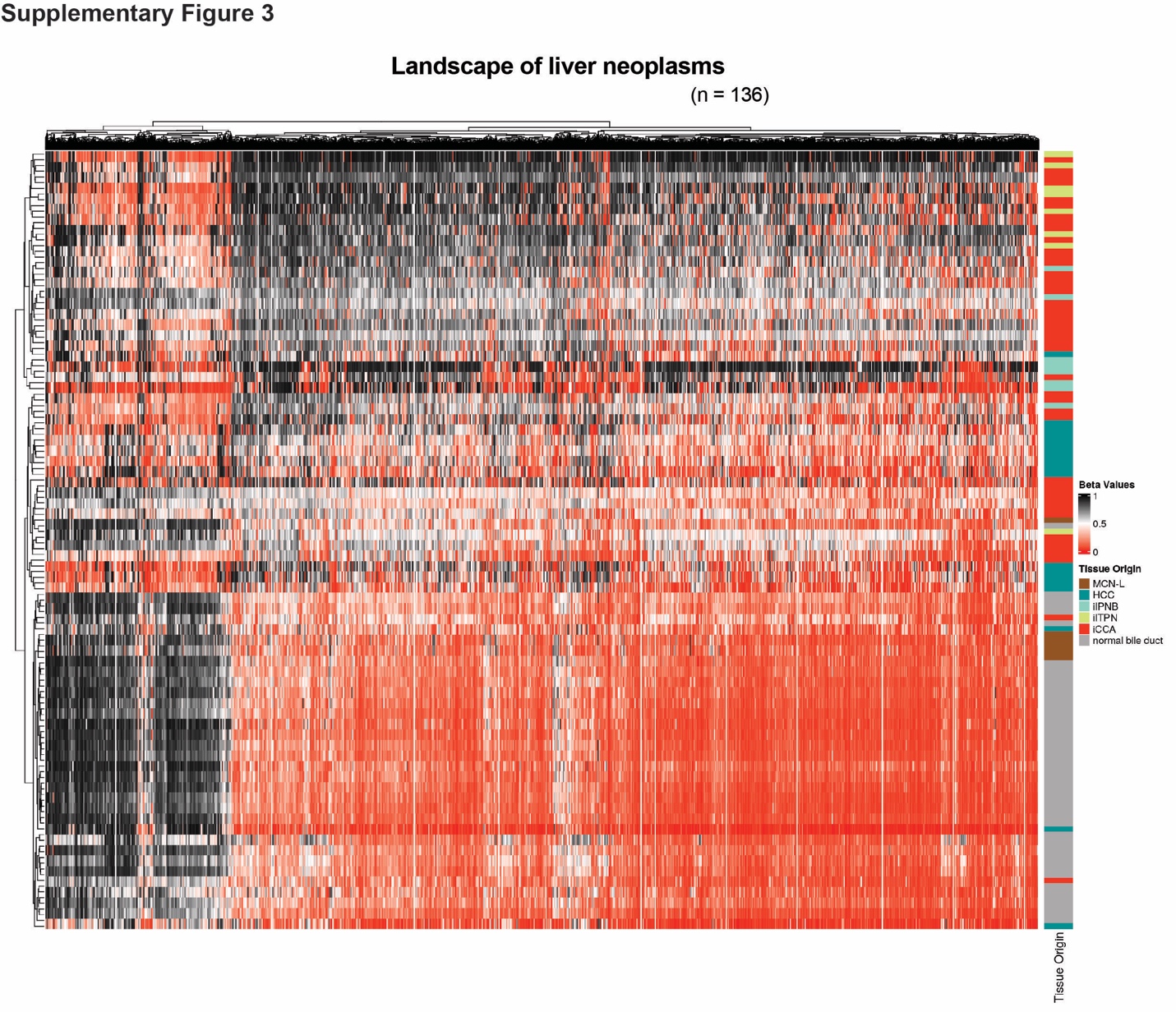
**

**Figure S3.** Hierarchical clustering of the samples composing the landscape of liver neoplasms.

**
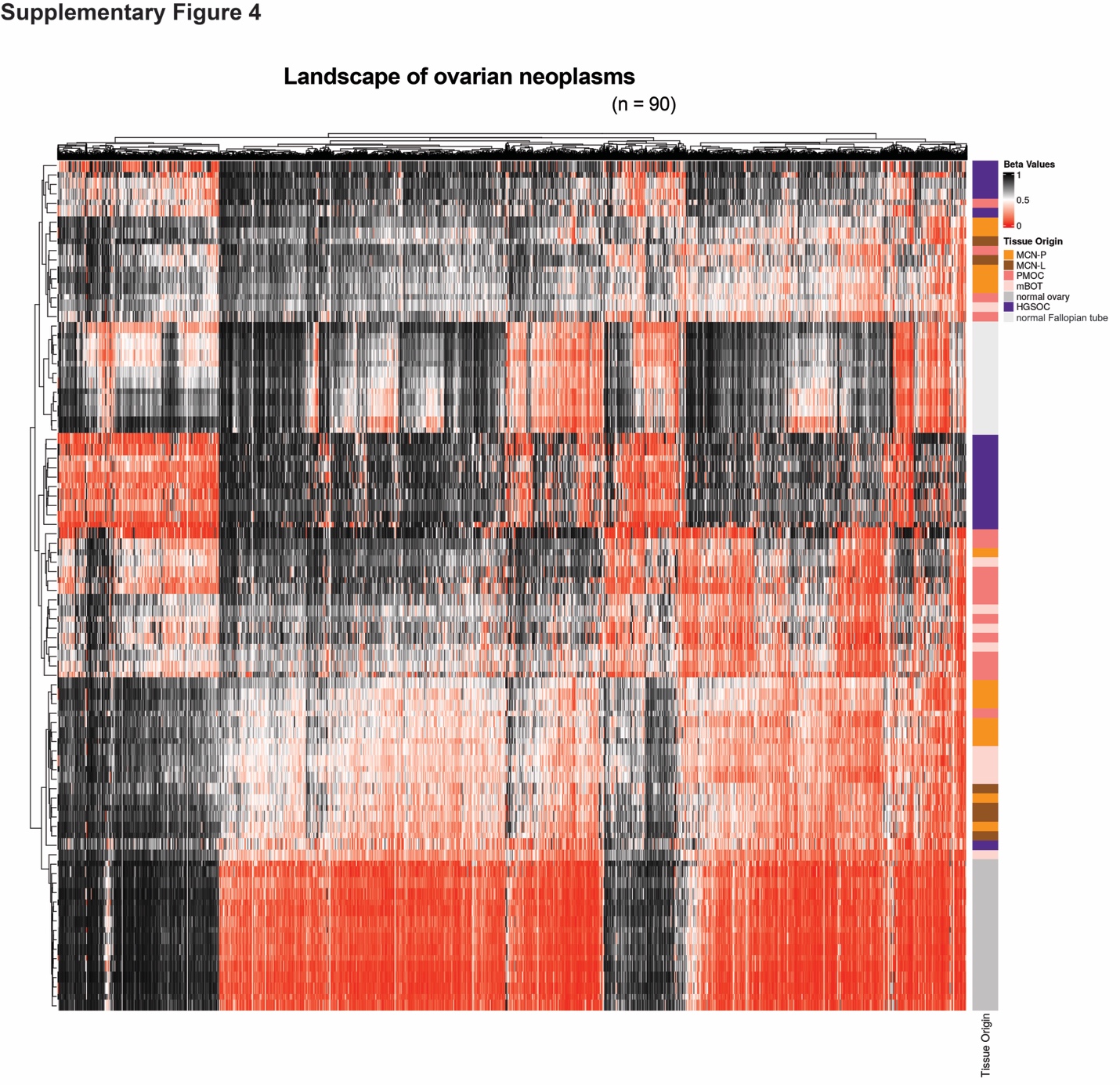
**

**Figure S4.** Hierarchical clustering of the samples composing the landscape of ovarian neoplasms.

**
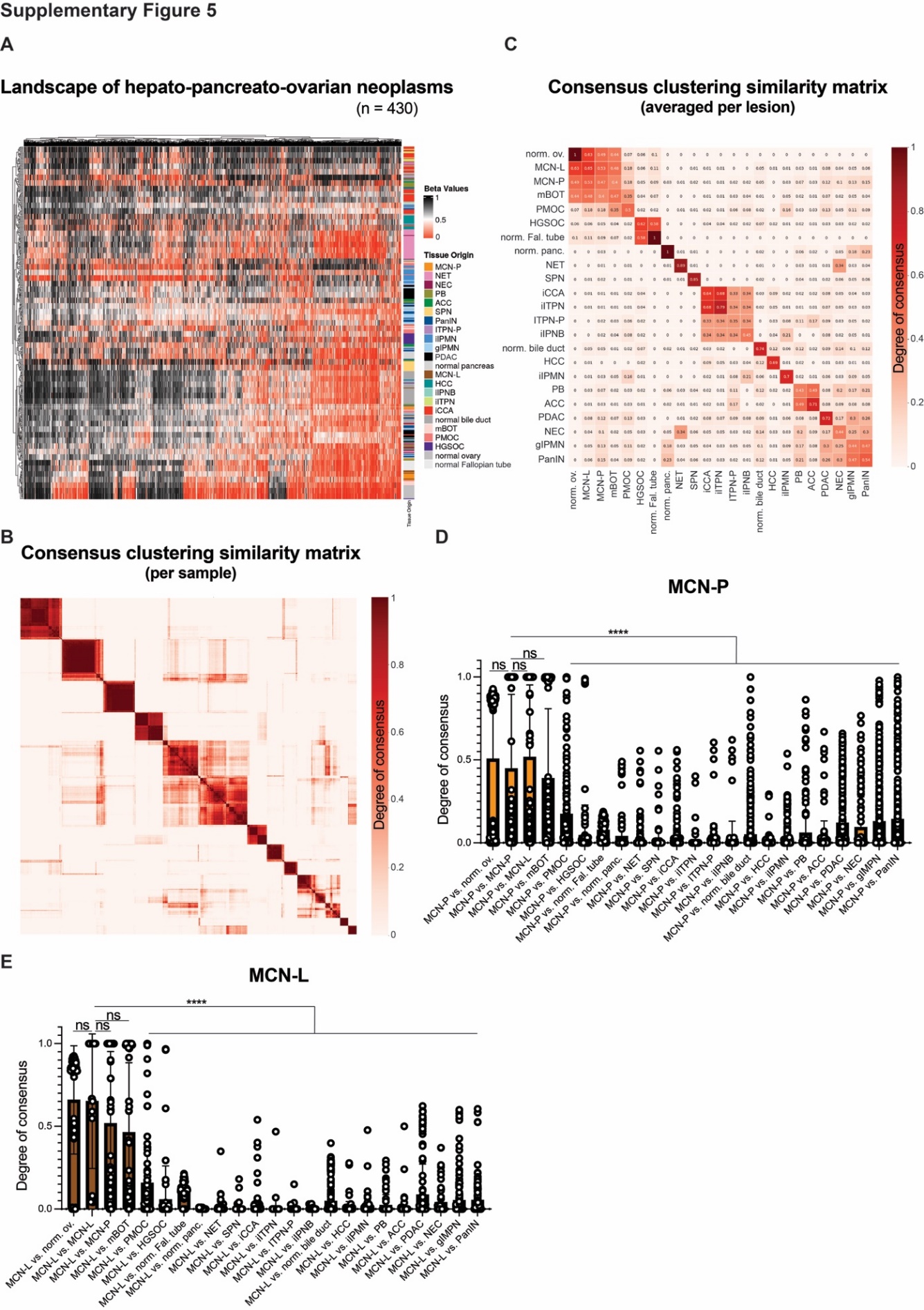
**

**Figure S5. Hierarchical and consensus clustering and sample clustering of hepato-pancreato-ovarian neoplasms.** (A) Hierarchical clustering of the samples composing the landscape of hepato-pancreato-ovarian neoplasms. (B) Per sample consensus clustering of each sample of the landscape of hepato-pancreato-ovarian neoplasms. The color code represents the degree of consensus for each sample. (C) Per group averaged consensus clustering of the samples composing the landscape of hepato-pancreato-ovarian neoplasms. The color code represents the average degree of consensus within a group. (D) Bar plots showing the degree of consensus within MCN-P samples versus all other groups separately. (E) Bar plots showing the degree of consensus within MCN-L samples versus all other groups separately. *****p* < 0.0001.

**
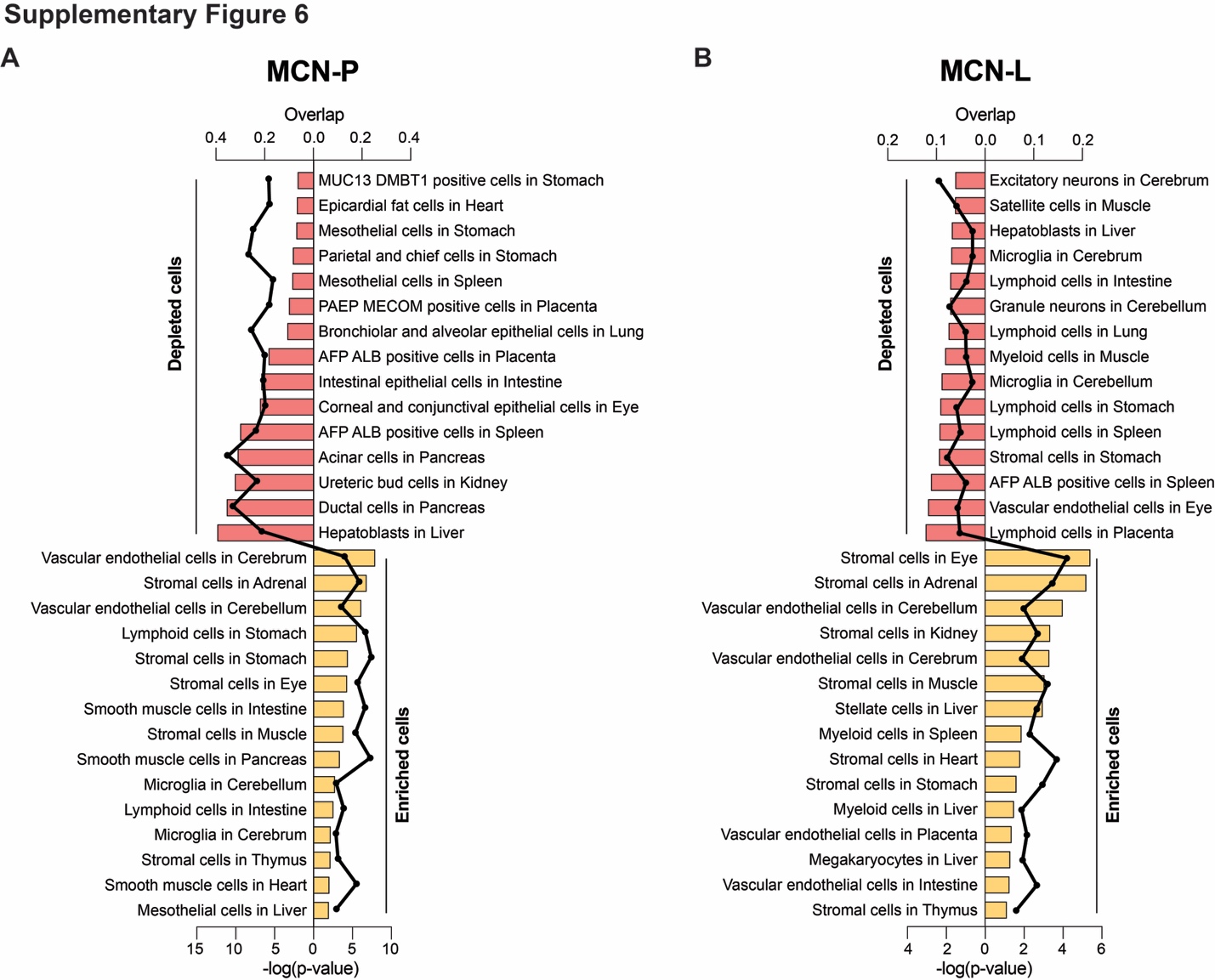
**

**Figure S6.** **Cell type enrichment analysis for genes linked to differential TSS methylation in MCN-P and MCN-L tissues.** (A) Top 15 cell types enriched and top 15 cell types depleted from the DESCARTES database for genes associated with the hyper- and hypo-methylated CpGs of TSSs in MCN-P versus normal pancreatic tissue. (B) Top 15 cell types enriched and top 15 cell types depleted from the DESCARTES database for genes associated with the hyper- and hypo-methylated CpGs of TSSs in MCN-L versus normal bile duct tissue. The bar plots represent the −log(*p* value) for cell type enrichment, and the connected dots represent the ratio of genes from the list matching the total number of genes specific for a given cell type.

**Table S1.** Characteristics of the antibodies used in immunohistochemical (IHC) analysis.

| **Antigen** | **Abbreviation** | **Clone** | **Origin** | **Manufacturer** | **Catalog No.** | **Dilution** | **Pretreatment method** |
| --- | --- | --- | --- | --- | --- | --- | --- |
| Cytokeratin 7 | CK7 | OV-TL 12/30 | Mouse | Dako/Agilent (Santa Clara, CA, USA) | M7018 | 1:1,000 | Protease 1 8 min |
| Cytokeratin 19 | CK19 | RCK108 | Mouse | BioGenex (Fremont, CA, USA) | MU246-UC | 1:100 | Protease 1 8 min |
| Cytokeratin 20 | CK20 | Ks20.8 | Mouse | Dako/Agilent | M7019 | 1:100 | Protease 1 8 min |
| Caudal type homeobox 2 | CDX2 | EPR276Y | Rabbit | Zytomed (Berlin, Germany) | RBK019-05 | 1:50 | CC1 mild |
| Carbohydrate antigen 19-9 | CA19-9 | C241:5:1:4 | Mouse | Leica (Newcastle upon Tyne, UK) | NCL-L-CA19-9 | 1:500 | Without pretreatment/ UltraView |
| Annexin A10 | ANXA10 | EPR19507 | Mouse | Abcam (Cambridge, UK) | ab213656 | 1:2,000 | Dewax, HP1 ER2 30 min |
| Mucin 1 | MUC1 (EMA) | E29 | Mouse | Dako/Agilent | M0613 | 1:100 | CC1 mild |
| Mucin 5AC | MUC5AC | 1G8 | Mouse | Leica | 18-2322 | 1:50 | CC1 mild |
| Chromogranin A | Chromo-granin A | EP38 | Rabbit | Epitomics (Burlingame, CA, USA) | AC-0037 | 1:100 | CC1 mild |
| Synaptophysin | Synapto-physin | 27G12 | Mouse | Leica | NCL-L-SYNAP-299 | 1:50 | CC1 mild |
| Estrogen receptor | ER | SP1 | Rabbit | Roche/Ventana (Tucson, AZ, USA) | 790-4325 | RTU | CC1 mild |
| Progesterone receptor | PR | 1 E 2 | Rabbit | Roche/Ventana | 790-4296 | RTU | CC1 mild |
| Neprilysin | CD10 | 56C6 | Mouse | Leica | NCL-L-CD10-270 | 1:50 | Dewax, HP1 ER2 30 min |

RTU, ready to use.

**Table S2.** Parameters used for quantitative expression analysis using QuPath version 0.5.1 (Queen’s University, Belfast, Northern Ireland) for epithelial and stromal immunohistochemistry markers.

| **Parameters for epithelial markers** | | **Parameters for stromal markers** | | |
| --- | --- | --- | --- | --- |
| Detection image | Optical density sum | Detection image | | Optical density sum |
| Pixel size | 0.2 µm | Pixel size | | 0.2 µm |
| Intensity threshold | 0.2 | Intensity threshold | | 0.2 |
| Background radius | 8 µm | Background radius | | 0 µm |
| Minimum area | 10 µm^2^ | Minimum area | | 10 µm^2^ |
| Maximum area | 400 µm^2^ | Maximum area | | 400 µm^2^ |
| Sigma | 1.5 | Sigma | | 1.5 |
| **Intensity threshold parameters epithelium** | | **Intensity threshold parameters stroma** | | |
| 1+ (weak) | 0.2 | 1+ (weak) | 0.1 | |
| 2+ (moderate) | 0.4 | 2+ (moderate) | 0.2 | |
| 3+ (strong) | 0.6 | 3+ (strong) | 0.4 | |

**Table S3.** Clinical and pathological characteristics and the DNA methylation array scan ID of the included samples.

| **Case No.** | **Gender** | **Age**  **(years)** | **Anatomical localization** | **Resection type** | **Tumor diameter (mm)** | **Low versus high grade + invasiveness** | **Scan ID** |
| --- | --- | --- | --- | --- | --- | --- | --- |
| 1 | F | 63 | Pancreas | Distal pancreatectomy | 70 | High and invasive | 207179230185_R01C01 |
| 2 | F | 40 | Pancreas | Cystectomy | 180 | High | 207179230144_R05C01 |
| 3 | F | 50 | Pancreas | Distal pancreatectomy | 89 | High | 207179230144_R02C01 |
| 4 | F | 35 | Pancreas | Distal pancreatectomy | 90 | High | 207331540101_R02C01 |
| 5 | F | 22 | Pancreas | Distal pancreatectomy | 60 | Low | 207331540050_R05C01 (did not pass technical check) |
| 6 | F | 52 | Pancreas | Distal pancreatectomy | 15 | Low | 207179230185_R04C01 |
| 7 | F | 34 | Pancreas | Distal pancreatectomy | 105 | Low | 207331540050_R06C01 |
| 8 | F | 50 | Pancreas | Distal pancreatectomy | 60 | Low | 207331540050_R07C01 |
| 9 | F | 36 | Pancreas | Cystectomy | 175 | Low | 207179230185_R03C01 |
| 10 | F | 30 | Pancreas | Distal pancreatectomy | 13 | Low | 207179230185_R06C01 |
| 11 | F | 41 | Pancreas | Distal pancreatectomy | 50 | Low | 207331540050_R02C01 |
| 12 | F | 35 | Pancreas | Distal pancreatectomy | 25 | Low | 207179230185_R05C01 |
| 13 | F | 61 | Pancreas | Distal pancreatectomy | 60 | Low | 207179230185_R07C01 |
| 14 | F | 60 | Pancreas | Distal pancreatectomy | 60 | Low | 207179230144_R04C01 |
| 15 | F | 31 | Pancreas | Distal pancreatectomy | 35 | Low | 207331540050_R03C01 |
| 16 | F | 64 | Liver | Partial hepatectomy | 62 | High and invasive | 207179230185_R02C01 |
| 17 | F | 53 | Liver | Partial hepatectomy | 85 | Low | 207179230144_R08C01 |
| 18 | F | 43 | Liver | Left hepatectomy | 110 | Low | 207331540050_R04C01 |
| 19 | F | 69 | Liver | Partial hepatectomy | 110 | Low | 207179230144_R06C01 |
| 20 | F | 57 | Liver | Partial hepatectomy | 110 | Low | 207331540050_R01C01 |
| 21 | F | 54 | Liver | Partial hepatectomy | 126 | Low | 207179230144_R03C01 |

All patients were female.

**Table S4**. H-scores of epithelial and stromal markers quantified using QuPath version 0.5.1 (Queen’s University, Belfast, Northern Ireland).

|  | **Epithelial** | | | | | | | | | | **Stromal** | | |
| --- | --- | --- | --- | --- | --- | --- | --- | --- | --- | --- | --- | --- | --- |
| **Case No.** | **CK7** | **CK19** | **CK20** | **CDX2** | **CA19-9** | **ANXA10** | **MUC1** | **MUC5AC** | **Chromo.** | **Synapto.** | **ER** | **PR** | **CD10** |
| 1 | 201 | 228 | 120 | 181 | 145 | 195 | 284 | 73 | 31 | 6 | 46 | 31 | 31 |
| 2 | 297 | 298 | 227 | 156 | 180 | 293 | 296 | 269 | 0 | 0 | 48 | 37 | 11 |
| 3 | 294 | 293 | 264 | 250 | 243 | 293 | 272 | 249 | 8 | 7 | 104 | 81 | 9 |
| 4 | 288 | 294 | 139 | 200 | 286 | 292 | 271 | 218 | 23 | 7 | 16 | 8 | 38 |
| 5 | 262 | 174 | 56 | 87 | 14 | 281 | 172 | 156 | 4 | 6 | 10 | 19 | 23 |
| 6 | 289 | 255 | 105 | 66 | 171 | 274 | 272 | 212 | 0 | 1 | 89 | 93 | 37 |
| 7 | 291 | 296 | 83 | 241 | 297 | 6 | 214 | 37 | 1 | 1 | 129 | 108 | 48 |
| 8 | 294 | 298 | 104 | 104 | 173 | 297 | 278 | 218 | 1 | 1 | 55 | 98 | 68 |
| 9 | 296 | 297 | 147 | 129 | 273 | 296 | 295 | 262 | 2 | 2 | 63 | 64 | 13 |
| 10 | 299 | 297 | 123 | 166 | 248 | 7 | 234 | 19 | 3 | 1 | 52 | 159 | 42 |
| 11 | 287 | 294 | 39 | 252 | 248 | 43 | 246 | 18 | 6 | 5 | 170 | 133 | 28 |
| 12 | 298 | 296 | 118 | 166 | 104 | 290 | 293 | 267 | 3 | 5 | 204 | 151 | 16 |
| 13 | 292 | 287 | 82 | 117 | 171 | 287 | 288 | 76 | 8 | 8 | 50 | 62 | 73 |
| 14 | 288 | 293 | 64 | 41 | 137 | 296 | 257 | 205 | 3 | 13 | 48 | 96 | 77 |
| 15 | 295 | 287 | 119 | 113 | 3 | 285 | 298 | 241 | 10 | 10 | 60 | 60 | 23 |
| 16 | 111 | 288 | 292 | 274 | 32 | 54 | 103 | 38 | 21 | 19 | 21 | 25 | 25 |
| 17 | 296 | 291 | 65 | 55 | 291 | 9 | 203 | 57 | 3 | 3 | 55 | 72 | 44 |
| 18 | 296 | 296 | 93 | 80 | 262 | 72 | 218 | 70 | 3 | 1 | 1 | 29 | 77 |
| 19 | 266 | 291 | 244 | 34 | 294 | 3 | 187 | 46 | 2 | 3 | 155 | 46 | 107 |
| 20 | 257 | 285 | 118 | 13 | 290 | 8 | 243 | 57 | 1 | 0 | 97 | 124 | 103 |
| 21 | 286 | 288 | 176 | 26 | 283 | 15 | 138 | 60 | 5 | 0 | 20 | 146 | 16 |

**Table S6.** Normal ovary cases: type of surgery, purpose of surgery, and histological description of the ovarian tissue.

| **Case No.** | **Age (years)** | **Type of surgery** | **Purpose of surgery** | **Histological description of ovary** |
| --- | --- | --- | --- | --- |
| 1 | 46 | Right oophorectomy | Ovarian tumor | Corpus luteum cyst |
| 2 | 44 | Laparoscopic leiomyoma morcellation and left oophorectomy | Leiomyoma of uterus | Corpus luteum cyst |
| 3 | 68 | Hysterectomy and bilateral oophorectomy | Uterine prolapse | No pathological changes |
| 4 | 32 | Hysterectomy and bilateral oophorectomy | Endometriosis | Follicle cysts |
| 5 | 39 | Partial myomectomy and left oophorectomy | Leiomyoma of uterus and benign ovarian cyst | Chronic salpingitis, follicle cysts, and inclusion cysts |
| 6 | 47 | Hysterectomy and right oophorectomy | Leiomyoma and endometriosis of uterus | No pathological changes |
| 7 | 67 | Hysterectomy and bilateral oophorectomy | Uterine prolapse | Inclusion cysts |
| 8 | 66 | Hysterectomy and left oophorectomy | Uterine prolapse and benign ovarian cyst | No pathological changes |
| 9 | 51 | Hysterectomy and bilateral oophorectomy | Leiomyoma of uterus | Follicle cysts |
| 10 | 72 | Hysterectomy and bilateral oophorectomy | Uterine prolapse | Follicle cysts |
| 11 | 47 | Bilateral oophorectomy | Suspected dermoid cyst | Atrophic ovary |
| 12 | 26 | Hysterectomy and bilateral oophorectomy | Ovarian cysts and cervical intraepithelial neoplasia | Follicle cysts |
| 13 | 53 | Hysterectomy and bilateral oophorectomy | Uterine prolapse | Follicle cysts |
| 14 | 78 | Hysterectomy and bilateral oophorectomy | Uterine prolapse | Inclusion cysts |
| 15 | 38 | Hysterectomy and right oophorectomy | Leiomyoma of uterus | Inclusion cysts and corpus luteum cyst |
| 16 | 49 | Hysterectomy and bilateral oophorectomy | Leiomyoma of uterus | Corpus luteum cyst |
